# Tracking the microbial communities from the farm to the processing facility of a washed-rind cheese operation

**DOI:** 10.3389/fmicb.2024.1404795

**Published:** 2024-08-29

**Authors:** Tara Wilson, Myra Siddiqi, Yueqi Xi, Gisèle LaPointe

**Affiliations:** Dairy at Guelph, Canadian Institute for Food Safety, Department of Food Science, University of Guelph, Guelph, ON, Canada

**Keywords:** milk quality, cheese, amplicon sequence variants, microbial ecology, sanitation, tap water

## Abstract

Milk residue and the accompanying biofilm accumulation in milking systems can compromise the microbial quality of milk and the downstream processes of cheese production. Over a six-month study, the microbial ecosystems of milk (*n* = 24), tap water (*n* = 24) and environmental swabs (*n* = 384) were cultured by plating decimal dilutions to obtain viable counts of total aerobic mesophilic lactose-utilizing bacteria (lactose-M17), lactic acid bacteria (MRS), yeasts and molds (Yeast, Glucose, Chloramphenicol (YGC) medium). Viable aerobic lactose-M17 plate counts of milk remained well below 4.7 log CFU/ml over five of the months, except for 1 week in November where milk at the facility exceeded 5 log CFU/ml. Swab samples of the farm milking equipment showed consistent viable counts after sanitation, while the bulk tank swabs contained the lowest counts. Viable counts from swabs of the facility were generally below the detection limit in the majority of samples with occasional residual contamination on some food contact surfaces. Extracted DNA was amplified using primers targeting the V3–V4 region of the 16S rRNA gene, and the amplicons were sequenced by MiSeq to determine the shared microbiota between the farm and the processing facility (8 genera). Culture independent analysis of bacterial taxa in milk, water and residual contamination after sanitation with swab samples revealed the shared and distinct microbiota between the sample types of both facilities. Amplicon sequence variants (ASVs) of the V3–V4 region of the 16S rRNA gene revealed that the microbiota of milk samples had lower diversity than water or environmental swabs (279 ASVs compared to 3,444 in water and 8,747 in environmental swabs). *Brevibacterium* and *Yaniella* (both *Actinomycetota*) were observed in all sampling types. Further studies will include whole genome sequencing of *Brevibacterium* spp. isolates to determine their functionality and diversity within the system.

## Introduction

In many large-scale cheese production operations, multiple sources of milk are standardized together before manufacturing. In contrast, on-farm operations usually use a sole source of milk. Contamination of milk can occur in the production environment indirectly from the transfer of microbes from the air, feed and bedding to the cow’s teats or directly from food contact surfaces and dairy equipment in the processing facility (Falardeau et al., 2019). The diversity of bacteria across both the production and processing environments may directly relate to the flavour development and aging profile of artisanal cheeses (Bokulich and Mills, 2013; Falardeau et al., 2019). Falardeau et al. (2019) specifically looked at the diversity of operational taxonomic units (OTUs) of bacteria between farm and finished cheese product, where *Firmicutes* were the most abundant phylum observed on both farm (31%) and finished cheeses (92%). Bokulich and Mills (2013) found that the cheese processing environment was dominated by fermentation-associated bacteria such as *Lactococcus* and *Debaryomyces,* which may aid in the development of cheese. D’Amico and Donnelly (2010) evaluated the microbial quality of raw milk collected from artisanal cheesemakers but focused predominately on the absence of four pathogens from the 21 artisan farms throughout their repeated sampling trials (D’Amico and Donnelly, 2010).

The raw milk microbiota contains a diverse consortium of bacteria which include dairy fermenters (*Lactococcus* and *Lactobacillus*) but can also be contaminated with foodborne pathogens such as *Salmonella* and *Listeria* (Quigley et al., 2013). A study by Van Kessel et al. (2004) investigated 861 bulk tank samples across 21 states and concluded that 95% of samples contained coliforms while on average, the SCC was determined at 295,000 cells/mL. Of these samples, only 2.5 and 6.5% contained *Salmonella* and *L. monocytogenes*, respectively, highlighting the sporadic nature of these latter contaminations. Good farm management practices are considered an influential factor for controlling undesirable microbes entering milk. Studies suggest that teat condition and teat contact areas are one of the highest contributors to bacterial contamination at the farm level but can be controlled with adequate cleaning procedures (Frétin et al., 2018; Du et al., 2020) and proper feed and bedding management. Storage and transportation of milk contribute to the raw milk microbial composition through equipment cleanliness, temperature, which may provide opportunity for microbial growth (O’Connell et al., 2016; Paludetti et al., 2018).

High-temperature-short-time (HTST) pasteurization is commonly applied before cheese manufacturing in North America. This technique quickly heats the milk to 72°C (161°F) for at least 15 s before being cooled to destroy bacteria which are sensitive to heat (Ibarrola et al., 1998). However, thermoduric and spore-forming bacteria, such as *Bacillus* sp. and *Clostridium* sp., may survive pasteurization, which can impact further processes (den Besten et al., 2018). Recent studies on the traceability of the raw milk microbiota using 16S rRNA gene amplicon sequencing and SourceTracker tool (Du et al., 2022) revealed that the main area of contamination could be the product holding tank. Studies in which tracking was done across the milk to cheese production found the microbiota of milk drastically changes from teat to storage tank (Falardeau et al., 2019) due to the accumulated selective pressures of temperature, time, and exposure to contamination.

The cheese production facilities could add possible sources of contamination and growth of microbes in milk. Even with the addition of starter and adjunct cultures, some studies suggest that artisanal ripening is based on the microbial ecosystem of the cheese production facility with little influence from the milk microbiota (Wolfe et al., 2014). Past studies have attempted to rationalize the batch-to-batch variations seen within the cheese production facility, where 25–41% variance was seen on food contact surfaces between facilities, and daily differences were attributed to the milk source and the contaminants accumulated from raw milk to thermized milk to vat milk (20% variance) (Johnson et al., 2021). Studies which investigated the role of food contact surfaces on the microbiota of the finished product found there were bidirectional interactions between biofilm producing bacteria from raw milk adhering onto cheese making tools such as wooden vats, resulting in the inoculation of the subsequent batch of raw milk (Sun and D’Amico, 2021).

Pasteurization and sanitation measures in production and processing facilities eliminate most pathogenic and spoilage bacteria. However, many bacteria can form biofilms, adhering to organic residue on surfaces such as stainless steel, which may reduce cleaning efficiency and effectiveness (O’Toole et al., 2003; Jefferson, 2004). Rosado de Castro et al. (2017) found that *Enterococcus faecium* and *Enterococcus faecalis* could form biofilms within 1–8 days of contact when the temperature ranged-between 12–47°C and 10–43°C, respectively. The reversible attachment step represents when the extracellular polymeric material is not yet formed and the bacteria are only weakly bound to the surface and can return to their planktonic state (Fu et al., 2021). During this attachment step, bacteria can effectively be removed by sanitizers (Rosado de Castro et al., 2017). Some of the most studied biofilm-forming organisms which can form biofilms in food production areas are *Escherichia coli*, *Pseudomonas aeruginosa*, *Bacillus subtilis*, and *Staphylococcus aureus* (Carrascosa et al., 2021). Environmental factors such as nutrient availability, antibacterial agents, temperature, and pH can induce biofilm formation in these organisms (López et al., 2010; Liu et al., 2020). Adhered microorganisms can pose a risk to dairy production due to the reduction in cleaning effectiveness, as biofilms can harbor pathogens and other contaminants, which increases the risk of residual contamination post-cleaning, leading to the release of bacteria to continue spreading through the production line (Flint et al., 2020). This can cause detrimental effects on downstream processes causing erosion, blockages, and insufficient heat transfer in dairy production equipment, which in turn can lead to the disruption of quality milk yields and cheese production (Seale et al., 2015).

Past studies have investigated the interconnection between farm and cheese facilities, mainly attributing the microbiota of the final cheese product to the initial microbiota found in raw milk (Falardeau et al., 2019; Sun and D’Amico, 2021; Du et al., 2022). However, most of these studies have been conducted over a short time span, whereas longitudinal studies can address the spatial and temporal variability of the system (Johnson et al., 2021). This study aimed to apply both culture-dependent methodology and 16S rRNA gene amplicon sequencing approaches to determine the occurrence and stability of post-cleaning residual contamination of a sole milk source cheese production facility over a six-month period in correlation with the microbial communities of raw milk and water. These results can help establish how temporal and spatial variance of these environments may influence the in-house microbiota of sole source cheese production.

## Methods

### Dairy production facility and cheese processing facility

The sampling was carried out on a dairy farm providing bovine milk to one cheese processing facility in Ontario, Canada, over the duration of six months. The dairy facility houses 120 pure Holstein dairy cows which are fed mixed rations consisting of grass, hay, and corn, producing an average of 2,000 L of milk per day. After milking, milk was cooled to 4°C in a bulk tank on the farm for 24 h, then was transported to the cheese production facility in a Dairy Farmers of Ontario regulated tanker vehicle. Milk was held in a cooled bulk tank at the cheese production facility until the next morning when milk was pasteurized at 72°C for 15 s. On the last two Tuesdays of each month over the duration of six months (June to November 2022), samples were collected from both facilities as described below.

### Collection of swab samples, raw milk, and water

**Figure 1:**
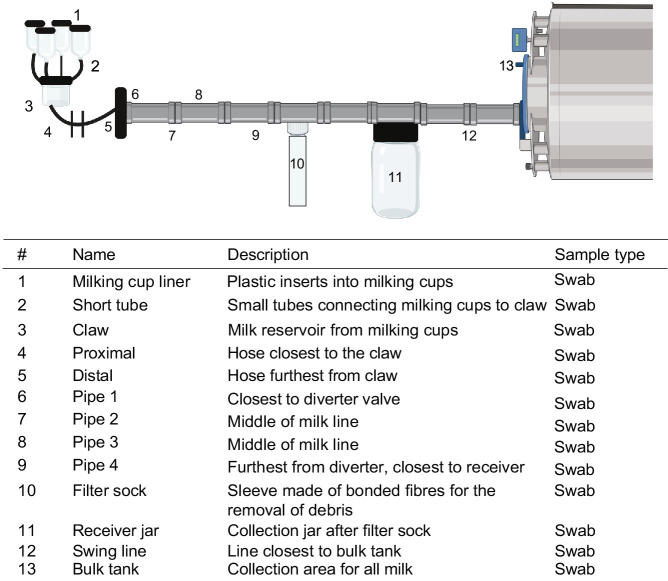
Flow schematic of sampling locations on farm. Numbers denote sampling locations within the farm environment with description and methods for collection listed. Swabs of hoses and pipes were taken by swabbing one side of the pipe 5 times for microbial methods and the opposite side for DNA extraction. Created using BioRender.com.

On the farm after milking and cleaning, duplicate sets of plastic cotton swabs which were moistened in saline were used to swab 13 areas of 10 cm^2^ or 5 half turns in the case of pipes (Figure 1). One of the duplicate swabs was stored in 10 mL of saline for microbial plating and the other was stored in 1 mL azidiol solution for DNA extraction. Twenty-five millilitres of milk were collected from the milk holding tank in each of two tubes, one containing 1 mL of azidiol solution for DNA extraction. All farm samples were stored at 4°C until transfer to the laboratory for further analysis. Samples were processed immediately upon receipt for microbial plating and cell pellets were collected as described below and stored at −20°C until DNA extraction.

**Figure 2:**
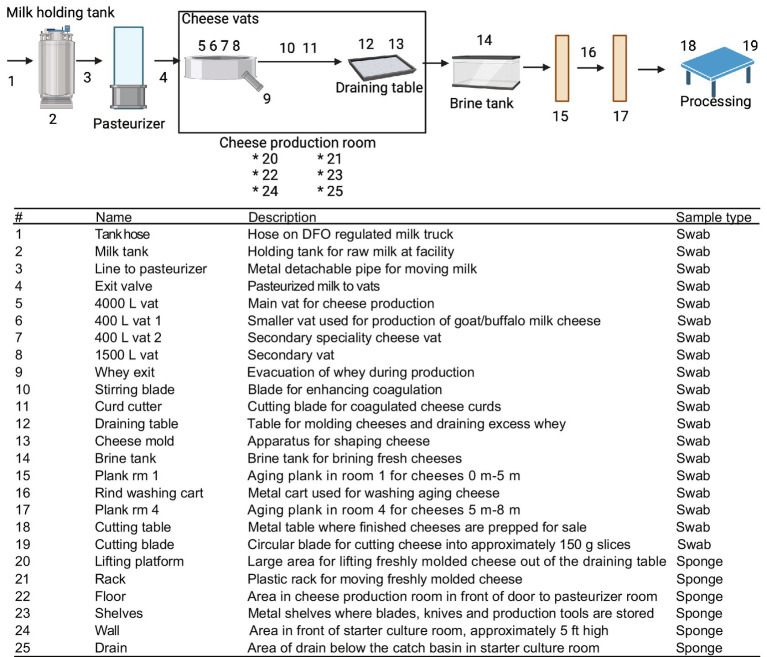
Flow schematic of sampling points within the cheese-making facility. Numbers denote sampling locations within the facility environment with description and methods for collection listed. Swabs of hoses and pipes were taken by swabbing one side of the pipe 5 times for microbial methods and the opposite side for DNA extraction, swab samples were taken in a 100 cm^2^ area and sponges over 1 m^2^. Created using BioRender.com.

Nineteen swab locations and six sponge locations were sampled at the cheese production facility after sanitation, with an additional 9 samples taken before sanitation which were used for DNA extraction (Figure 2). Duplicate dry sponge samples were obtained from larger surface areas; floors, walls and drains in a 1 m^2^ area and stored in sterile bags. Duplicate sets of swabs were obtained from all 19 locations, one for microbial plating and the other for DNA extraction. Water samples were obtained in the starter culture room by flushing the system for 45 s before collecting a volume of 2 L from the tap in each of two plastic bottles. For both facilities, water is supplied through groundwater wells which are treated with chlorine. All samples were held at 4°C until further analysis at the laboratory.

### Viable counts of bacteria, yeast, and mold

For samples obtained from the dairy facility, swabs were vigorously vortexed for 30 s before 1 mL of liquid sample was used for serial dilutions in 9 mL of peptone-saline solution (8.5 g/L NaCl and 1 g/L BactoPeptone). A volume of 100 μL of undiluted suspension was also plated in duplicate. Sponges premoistened in Letheen broth (3 mL) were used for larger surface areas (wall, floor, racks) and were homogenized by hand in 27 mL of peptone saline before plating 100 μL of undiluted duplicates. From one of the tubes containing twenty-five millilitres of milk collected, 1 mL was serially diluted in 9 mL of peptone-saline solution, and 100 μL of undiluted milk was also plated in duplicate. Each of the 2-L water samples was filtered through a 0.22 μm Fisher bottle top filter, the filters were removed, cut and suspended in 3 mL of peptone-saline then vortexed for 45 s. For one of the filtered water suspensions, a volume of 100 μL of the suspension was plated in duplicate, and 1 mL of suspension was transferred for serial dilution in 9 mL of peptone-saline and plated in 100 μL duplicates. All samples were plated onto two media types; M17 agar supplemented with 0.5% lactose (LM17) for enumeration of total aerobic mesophilic lactose-utilizing bacteria (Terzaghi and Sandine, 1975) and de Man Rogosa Sharpe agar (MRS) supplemented with 1% vancomycin (MRS-V) under anaerobic conditions for non-starter lactic acid bacteria, mainly *Lactobacillus* spp.. Samples were incubated at 30°C for 72 h before counting colonies on plates with between 10 and 200 total colonies (Jongenburger et al., 2010). This gives a lower limit of quantification (LOQ) of 2.2 log CFU per L for undiluted water samples, 2.0 log CFU per mL for undiluted milk samples, brine and rind wash water samples, 3.0 log CFU per swab for undiluted swab samples and 3.5 log CFU per sponge for sponge samples. Samples collected from the cheese facility were also plated on Yeast, Glucose, Chloramphenicol (YGC) medium incubated aerobically at 25°C for 72 h for the enumeration of yeasts and molds.

### DNA extraction

Swab samples were vortexed for 30 s before expelling the liquid and then aseptically removing the swab. Solution expelled from swabs of the claw, cup, liner, short hose, proximal and distal hoses of the milking system were pooled into one cell pellet for DNA extraction. Similarly, the solution expelled from swabs of pipelines 1–4 were pooled into one cell pellet for DNA extraction. A volume of 10 mL of the sponge suspension was collected for centrifugation to obtain the cell pellet for DNA extraction. The duplicate water filter membrane suspension was transferred to a new tube for centrifugation to obtain the cell pellet for DNA extraction. DNA from cell pellets collected from swab and sponge suspensions and the water filter was extracted using Qiagen DNeasy PowerSoil Pro Kit (Cat No. 47014) as per the manufacturer’s protocol. As modified from Quigley et al. (2012), 10 mL of milk were suspended in 90 mL of 2% (wt/vol) trisodium citrate dehydrate warmed to 45°C and homogenized for 5 min at 260 rpm using Seward Stomacher^®^ 400 Circulator. After homogenization, 10 mL of homogenate was transferred into a 15 mL falcon tube and centrifuged for 10 min at 10,000 x *g*. Sterile cotton swabs were used to remove residual fat and supernatant. The pellet was washed twice with 2% (wt/vol) sodium citrate dehydrate before the milk cell pellet was resuspended in PowerBead solution as per the manufacture instructions for Qiagen DNeasy UltraClean microbial kit (Cat No. 10196). After extraction, DNA quantity and quality was measured by Nanodrop and Qubit (1x dsDNA HS Assay Kit) to determine the DNA concentration of each sample.

### 16S rRNA gene amplicon sequencing

Purified DNA (over 5 ng/μL; see Supplementary Tables S1–S3) from milk, swab, and water samples was sent to the Advanced Analysis Centre (AAC) at the University of Guelph, Guelph, Ontario, Canada for sequencing of the V3–V4 region of the 16S rRNA gene amplicon using the Illumina MiSeq platform (Illumina, San Diego, CA, United States). The method, as described in Barzideh et al. (2022), was used with some modifications. Before library preparation, 10 μL of DNA was adjusted to 5 ng/μL concentration. Amplicon sequencing libraries were prepared following the 16S Metagenomics Sequencing Library Preparation Guide with some modifications (Illumina 2020). The V3 and V4 region (~460 bp) of the 16S rRNA gene were amplified using the following universal primers: Forward primer: 5’ CCTACGGG NGGCWGCAG and Reverse primer: 5’GACTACHVGGGTATCTAATCC (Klindworth et al., 2013). After library preparation, the purified amplicons were combined in equal molar ratios based on their DNA concentrations. The pooled libraries were denatured with 1 mM NaOH, diluted with hybridization buffer, and then heat denatured prior to sequencing. An internal control of PhiZ was included at a 15% level. Sequencing was conducted using the MiSeq sequencer with the MiSeq v4 reagent kit and 2 × 300 paired-end cycles according to the manufacturer’s protocol. All raw sequence reads were filtered using the Miseq Sequencer System software to remove low quality sequences and trimmed to remove the adapter sequences. The resulting reads were up to 301 bases long.

### Data analysis

All 16S rRNA gene amplicon sequence data was analyzed using R studio for amplicon sequence variants (ASVs). Forward and reverse reads were merged, and chimeras deleted using DADA2. All samples were trimmed at 285 b forward and 200 b reverse to remove primer sequences and samples with less than 500 reads were excluded from further analysis. Twenty-two samples from the farm and 7 samples from the facility were removed due to low read counts (<500). Filtering was first carried out at the phylum level at 80% prevalence in all samples and 0.001% abundance to identify potential shared phyla between milk, water and swabs, which resulted in the identification of *Actinomycetota* (synonym *Actinobacteria*) as the only shared phylum present in over 80% of samples. Filtering was then set at 20% prevalence, meaning that genera which were identified in at least 20% of all samples and above 0.01% relative abundance were retained for community profiling. For ASV analysis, amplicon sequence variants were classified using SILVA version 138.1 (McLaren and Callahan, 2021). LEfSE analysis was used to identify significantly different ASVs across spatial sampling sites. Significant differences between month (June–November), and location type (farm versus facility) were determined using Tukey’s Honestly Significant Difference (HSD) pairwise comparisons. Variance was based on eigenvalues from principal coordinate analysis (PCoA) representing the variance across the first two components. Figures and tables were generated in Excel and Microbiome Analyst.

## Results

### Culture dependent methods

#### Viable counts of bacteria in raw milk

The LM17 counts during the months of August and September trended higher than other months, but the difference was not significant (Table 1). The LM17 count was similar between the raw milk on farm and the cheese facility, with only small deviations (below 1 log). Tukey’s HSD revealed no significant differences between location or month, indicating the stability of the bacterial load of milk during transfer over the duration of the study (Pr > F 0.521). During the month of November, the culturable bacteria of raw milk at the facility reached over 5 log CFU /mL on LM17. Further testing of November milk on 3 M™ Petrifilm *E. coli*/Coliform Count Plates showed a count of 3 log CFU/mL in raw milk, but coliforms were absent from fresh cheese curd made from the pasteurized milk.

**Table 1:** Viable counts of bacteria (log CFU per ml on LM17 medium and MRS-V medium) in raw milk from the farm bulk tank and from the cheese facility.

Average viable counts of bacteria (log colony forming units (CFU) / mL ± log standard deviation)							
Sample	Media type	June	July	August	September	October	November
Farm	LM17	2.78 ± 0.16	2.50 ± 0.34	3.28 ± 0.66	3.11 ± 0.09	2.84 ± 0.08	2.65 ± 0.15
	MRS-V	2.46 ± 0.10	2.78 ± 0.46	2.11 ± 0.10	2.81 ± 0.32	2.83 ± 0.54	2.36 ± 0.07
Facility	LM17	2.62 ± 0.30	2.63 ± 0.27	2.98 ± 0.16	3.09 ± 0.12	3.31 ± 0.32	4.77 ± 1.96
	MRS-V	2.47 ± 0.13	<2.0	2.39 ± 0.42	<2.0	2.84 ± 0.08	2.64 ± 0.60

There were no significant differences found between location and/or month. The lower limit of quantification for the plate count method was 2.0 log CFU per ml. LM17 medium was incubated aerobically at 30°C for 72 h, while MRS-V medium was incubated anaerobically at 30°C for 72 h.

#### Viable counts of bacteria in water from farm and facility

Culturable bacteria of tap water show similarities between water collected from the farm and from the cheese processing facility. For both facilities, water contained viable counts on MRS-V in only one of the 6 months (Farm in June and Facility in August). Very few counts were seen on MRS-V so that data is not shown for water. Culturable bacteria enumerated on LM17 varied slightly by month and between farm and facility (Table 2), but with no significant differences. Viable counts of water samples collected from the farm in August were below the limit of quantification (LOQ 2.2 log CFU per L), in comparison to water obtained from the facility which showed 4 log CFU/L for the same month.

**Table 2:** Average viable count of bacteria (log CFU per L plated on LM17 incubated aerobically) in water from the farm and the cheese facility.

Average viable count of bacteria (log colony forming units) (CFU) / L ± log standard deviation			
Month	Week	Farm	Facility
June	W1	<2.2	3.65 ± 0.001
	W2	<2.2	3.46 ± 0.01
July	W1	<2.2	4.49 ± 0.01
	W2	3.51 ± 0.01	3.67 ± 0.24
August	W1	<2.2	3.19 ± 0.002
	W2	4.03 ± 0.07	4.30 ± 0.04
September	W1	<2.2	2.86 ± 0.01
	W2	3.66 ± 0.03	3.34 ± 0.03
October	W1	<2.2	4.64 ± 0.01
	W2	2.27 ± 0.14	2.87 ± 0.15
November	W1	2.91 ± 0.06	<2.2
	W2	<2.2	2.61 ± 0.16

For the values above LOQ, there were no significant differences found between location and/or month. The lower limit of quantification of the plate count method for water samples was 2.2 log CFU/L.

#### Viable counts of bacteria from swab samples from farm and facility

**Figure 3:**
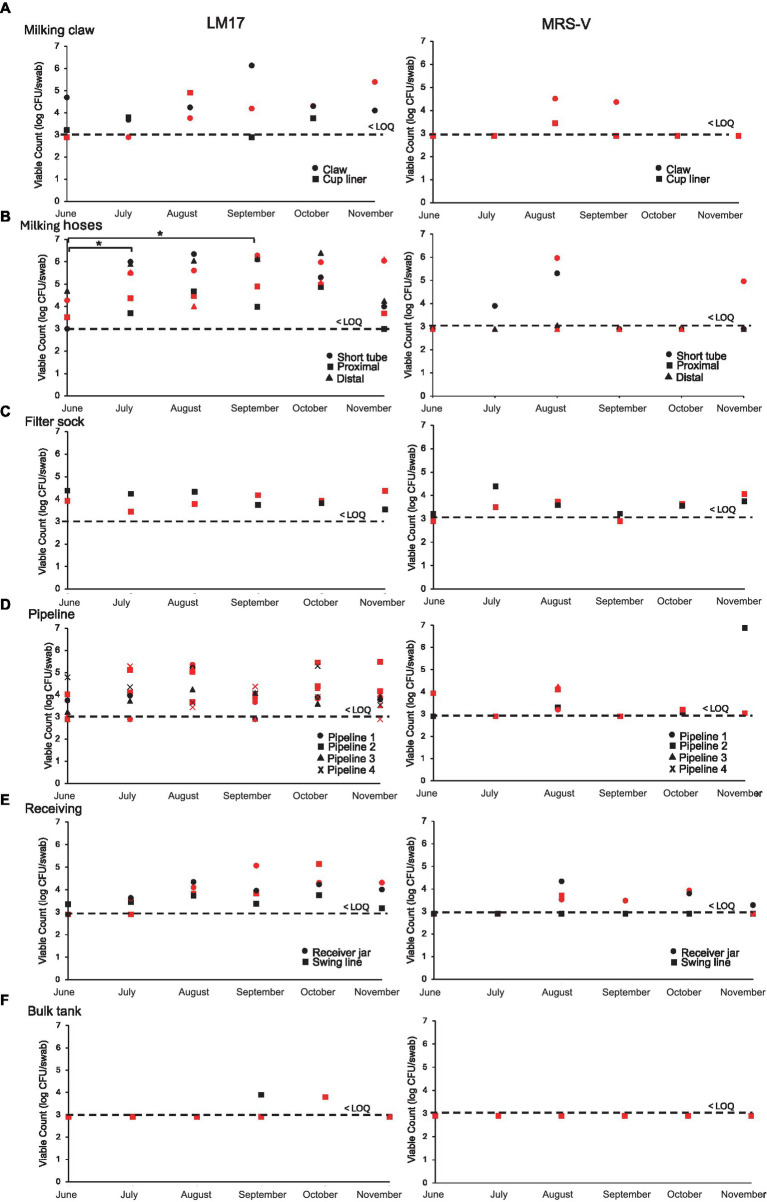
Viable plate counts (log CFU per swab) obtained on LM17 and MRS-V media for environmental samples obtained from the farm over 6 months. **(A)** Claw and cup liner, **(B)** Hoses, **(C)** Filter sock, **(D)** Pipeline, **(E)** Receiver, **(G)** Bulk tank. Sample type is denoted in each plot by shape, red shapes indicate samples obtained in week 1, black shapes indicate samples obtained in week 2 of each respective month. The dashed line indicates the lower limit of quantification (LOQ) for swab samples obtained from the farm and is calculated at 3 log CFU per swab. All samples below LOQ are placed at the LOQ line. Significance at *p* < 0.05 is represented between months by an asterisk (* in **B**).

A total of 13 swab locations from the farm were sampled each week and plated on LM17 and MRS-V (Figure 3). The LM17 counts of both the cup liner and the claw were more often above than below the limit of quantification of 3.0 log CFU per swab, reaching 6 log CFU/swab for the claw. Following the claw, the LM17 counts of milking hoses from July to November were consistently recorded above the LOQ up to 6 log CFU/swab, but the counts on MRS-V were more often below the LOQ. This is then followed by the filter sock, which, on average, ranged between 3.5 and 4.5 log CFU/swab of culturable bacteria. The milking pipelines, which were sampled in four separate locations, showed counts on LM17 ranging from <LOQ to 5 log CFU per swab. MRS-V plate counts showed milking pipeline 2 in November approximating 7 log CFU/swab of viable bacteria. Culturable bacteria in the receiving jar showed an increasing trend over the months, beginning in June at <LOQ, rising to 5 log CFU/swab near the end of the sampling period in October and falling slightly (~1 log) in November. The counts from the bulk tank were significantly lower than the rest of the sampling locations (Tukey’s HSD *p* < 0.05), only exceeding LOQ at 2 sampling times. The LM17 counts in hoses (short tube, proximal and distal milk hoses) were significantly higher than the rest of the sample groups by Tukey’s HSD (*p* < 0.05).

**Figure 4:**
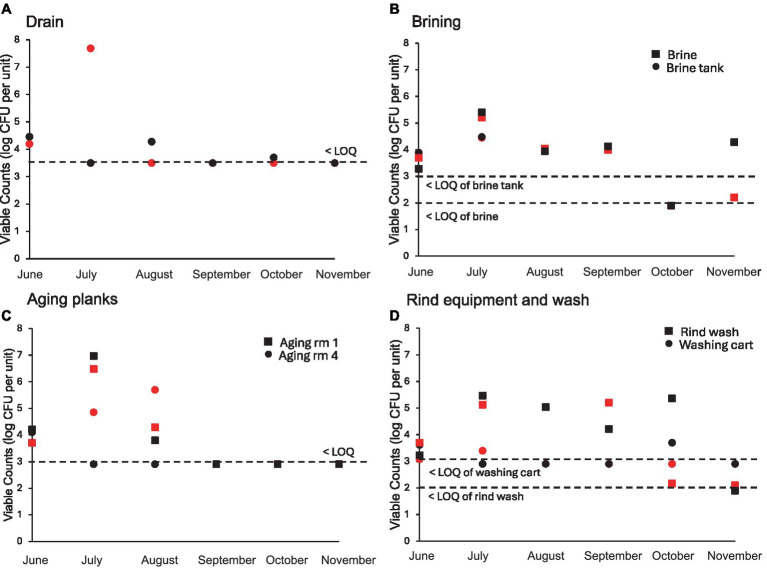
Individual viable plate counts (log CFU per unit) obtained on LM17 media for environmental samples obtained from the cheese facility (surfaces after sanitation except for brine tank and planks). **(A)** Drain (log CFU/sponge), **(B)** Brining equipment (log CFU/swab and log CFU/L), **(C)** Cheese aging planks (log CFU/swab), **(D)** Rind washing equipment (log CFU/swab and log CFU/mL). Sample type is denoted in each plot by shape, red shapes indicate samples obtained in week 1, black shapes indicate samples obtained in week 2 of each respective month. The lower limits of quantification (LOQ) are: 3.5 log CFU per sponge (Drain), 3.0 log CFU per swab (brine tank and aging planks), 2.0 log CFU per mL for the brine and rind wash solutions.

A total of 25 sites were sampled from the cheese making facility after cleaning and sanitation each month on 1 day in each of two successive weeks (Figure 4). Nine samples were obtained before sanitation in the months of June, July, and October, where the viable count on LM17 did not exceed 4 log CFU/swab. Food contact surfaces after sanitation showed only occasional viable counts exceeding LOQ (3 samples in June and July which included the milk tank once and the cheese cutting surface twice after sanitation where finished cheeses are cut before wrapping and sale). Viable counts of swabs from pipes within the facility did not exceed the LOQ (3.0 log CFU/swab). The tanker hose from the milk truck was collected three times (June, July, and August) before milk was transferred into the holding tank at the cheese making facility. The LM17 bacterial counts on two occasions exceeded the LOQ (June and July) but remained below 4 log CFU/swab. Among sponge samples (all of 1 m^2^), the floor samples showed the highest bacterial accumulation (between 4 and 5 log CFU/sponge in June and July). The viable counts of the wall and lifting platform were all below the LOQ over the 6 months (data not shown). The drain in the starter culture room (Figure 4A) showed the greatest weekly variability of all sampling sites, due to the daily rinsing, with biweekly thorough cleaning schedule of this location. The drain was significantly different from all other sample types (Tukey’s HSD *p* < 0.05). In some months (i.e. July), counts on LM17 were high (~7.5 log CFU/sponge) in the first week of sampling compared to counts below the LOQ in the second week, reflecting the biweekly deep cleaning schedule before the second week sampling. For the remaining months, the viable counts in the drain lie closer to each other, indicative of the similar microbial loads. Next, the brine solution was replaced in the tank early in the year (February) then again in August. This explains the highest bacterial accumulation seen in July with the brine nearing the end of the holding period.

Cheeses are kept on the same board throughout aging, beginning in room 1 (fresh cheeses at 0 m) then moving to room 4 at 5 m until final sale at 8 m. Cheeses were washed every 2–3 days in Room 1, but only every 3–5 days in Room 4, with separate salt solutions. Due to this washing schedule, planks with freshly washed cheeses may have higher viable counts on LM17 than planks with cheeses that were washed earlier. Planks sampled in aging room 1 with younger cheeses showed higher bacterial counts than planks holding older cheeses in Room 4 (*p* < 0.05), perhaps because they were washed more frequently. As expected, the rind wash (used for Room 4 cheese only) which was obtained during cheese washing had high bacterial accumulation, averaging around 5 log CFU/mL each month. The wash cart was sampled after cleaning using detergent and hot water, where the viable counts were generally below LOQ, except in June (3 and 4 log CFU/swab on LM17).

#### Viable counts of yeast and molds from the cheese facility

The yeast and mold count remained below the detection limit for 17 out of the 25 samples including tap water samples. Sporadic contamination of pipes, such as the line to the pasteurizer, was seen in 1 week of June, October, and November (Table 3). The drain had consistent fungal counts in all months other than August. The brine tank showed one count of 4.33 log CFU/swab during 1 week in July just before the brine was replaced and the tank cleaned. As expected, planks in aging room 4 showed high fungal accumulation in 4 out of 6 months, but in at least one of the weeks of September and November, had viable counts that were lower than the LOQ, which may have been due to the rind washing schedule. The rind washing water ranged from below LOQ in July up to 6.50 log CFU/mL of yeast and mold in September. Brine counts ranged from 3.58 to 6.50 log CFU/mL, except for counts below the LOQ in at least one of the weeks during June, July, September and October. Yeast and mold counts of milk ranged from 2.63 to 3.93 log CFU/mL.

**Table 3:** Viable yeast and mold count on YGC agar incubated aerobically for all sampling types within the facility.

Viable yeast and mold count (log CFU per unit* ± standard deviation)							
Type	Location	June	July	August	September	October	November
Swab	Tank hose	<3.0	<3.0	–	–	–	–
	Milk tank	<3.0	<3.0	<3.0	<3.0	<3.0	<3.0
	Line to pasteurizer	<3.0	<3.0	<3.0	<3.0	3.43 ± 0.02 ^2^	<3.0
	Stirring blade	3.45 ± 0.03 ^1^	<3.0	<3.0	<3.0	<3.0	<3.0
	Draining table	<3.0	<3.0	<3.0	3.24 ± 0.18 ^1^	<3.0	<3.0
	Brine tank	<3.0	4.33 ± 0.01^1^	<3.0	<3.0	<3.0	<3.0
	Plank rm. 4	>5.0	4.63 ± 0.02 ^2^	>5.0	<3.0	5.65 ± 0.75	<3.0
	Rind washing cart	<3.0	<3.0	<3.0	<3.0	<3.0	<3.0
	Cutting table	<3.0	<3.0	<3.0	<3.0	<3.0	<3.0
	Drain	3.45 ± 0.12 ^2^	4.15 ± 0.05 ^1^	<3.0	4.64 ± 0.51	3.48 ± 0.30	5.19 ± 0.79
Milk	Reception (mL)	3.53 ± 0.08 ^2^	–	–	2.63 ± 0.24	2.89 ± 0.08	3.93 ± 1.60
Brining	Brine (mL)	3.85 ± 0.06 ^2^	<2.0	5.01 ± 0.02	6.50 ± 0.07 ^2^	3.58 ± 0.04 ^2^	4.75 ± 0.44
	Rind wash water (mL)	3.53 ± 0.08 ^2^	3.45 ± 0.07 ^2^	<2.0	6.22 ± 1.40	5.65 ± 1.35	–

(−) A dash denotes that no sample was obtained for that month; * Units are in log CFU per swab, per mL for milk and wash water and brine. The lower limit of quantification for swabs was 3.0 log CFU per swab, 2.0 log CFU per mL was the lower detection limit for brine and rind wash water. ^1^ Average count for week 1 only; ^2^ Average count for week 2 only. No samples were significantly different over the duration of study based on Tukey’s HSD.

### Culture independent methods

#### 16S rRNA gene amplicon community profiling of raw milk samples

In total, 24 raw milk samples were collected over the six-month period. Fourteen out of 24 samples (58%) contained adequate DNA concentration (~5 ng/μL) for 16S rRNA gene amplicon sequencing (Supplementary Table S1). Five of these 14 samples were obtained from the farm (42% of farm milk samples), and 9 were from the facility (75% of facility milk samples). In total, 279 ASVs were found in milk ranging from 500 to 32,787 reads per sample (Supplementary Figures S1, S4). Before prevalence filtering, a total of 44 genera were distinguished. Taxa of *Pseudomonas*, *Lactiplantibacillus, Limosilactobacillus, Enterobacter, Staphylococcus, Stenotrophomonas,* and *Paeniclostridium* were identified at low prevalence (<20%) and 0.01% relative abundance (data not shown). After filtering at 0.01% relative abundance and 20% prevalence, seven genera remained: *Brevibacterium*, *Paracoccus, Kocuria, Enhydrobacter, Knoellia, Yaniella* and *Rothia*. While the relative abundance of *Enhydrobacter* spp. was significantly higher in the facility (*p* < 0.05), the five other genera trended higher in relative abundance on farm compared to the cheese facility but with no significant differences (*p* > 0.05). *Brevibacterium* represented the greatest relative abundance seen on farm (~50% RA), followed by *Paracoccus* and *Kocuria* (~40% RA)*. Kocuria* (30% RA) was the most abundant genus found in the facility, followed by *Enhydrobacter* (25% RA). Non-metric dimensional scaling (NMDS) showed only minor dissimilarities in beta diversity of the microbial communities between the farm and cheese processing facilities, as the *p*-value was not significant from the PERMANOVA analysis (Supplementary Figure S5). Only two sampling months from the facility (July and August) fell outside of the farm grouping, highlighting the similarity between the farm and cheese facility microbial populations found in milk for most months. The variance in beta diversity not accounted for by the first two components can be approximated at 85% for milk samples obtained from the farm and facility (calculated from PCoA analysis).

#### 16S rRNA gene amplicon community profiling of tap water

**Figure 5:**
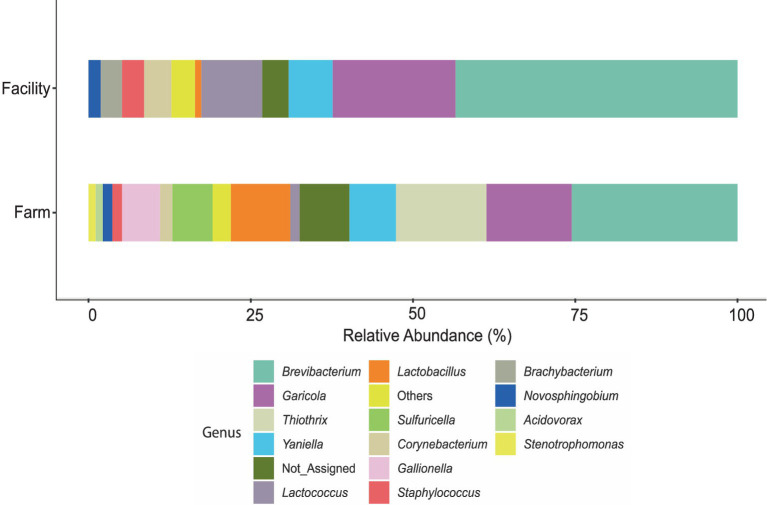
Stacked bar plot of the relative abundance of genera obtained from ASV analysis on DNA extracted from water samples from the farm and cheese production facility. Total ASVs were summed for each genus to determine relative abundance in the respective sampling area. The “Others” category represents samples which contained taxa with less than 500 reads after filtering.

Ten out of 24 tap water samples collected over the trial period provided adequate DNA (superior to 5 ng/μL) for 16S rRNA gene amplicon sequencing (Supplementary Table S2), and sequencing reads ranging from 1,909 to 127,818. Seven of these samples were obtained from the farm, whereas three were obtained from the cheese facility. Unfiltered data revealed a total of 3,444 ASVs in water samples from farm and facility. After filtering at 20% prevalence and 0.01% relative abundance, a total of 1,568 ASVs remained. Taxa which were below 500 reads after filtering were merged and are denoted as “Others.” Bar plots of the identified genera show some similarities and grouping of profiles between the two facilities (Figure 5; Supplementary Figure S2). *Brevibacterium* spp. was observed in high abundance (32%) in water samples obtained from the cheese facility. *Brevibacterium* was also the genus with the highest abundance on farm but was not significantly different from the abundance in the facility determined by Tukey’s HSD (Pr > Diff 0.495). Eleven genera were identified in the facility, compared to 15 on farm. Eight genera were shared between water from both facilities and were identified as: *Brachybacterium, Staphylococcus, Corynebacterium*, *Lactobacillus, Lactococcus*, *Yaniella, Garicola,* and *Brevibacterium.* The families of *Brevibacteriaceae* and *Micrococcaceae* comprised the main fraction of the relative abundance of bacteria in water (almost 50% on farm, 70% in the facility; Supplementary Figure S6). Genera which were unique to the farm water include: *Sulfuricella, Thiothrix, Acidovorax, Stenotrophomonas,* and *Gallionella. Novosphingobium* was the unique genus was found in the cheese facility. In total, there were 371 ASVs (23.6% of all ASVs) which were Not_Assigned (N/A) to genus level using the Silva database. After conducting a search using the Basic Local Alignment Search Tool (BLAST) on Not_Assigned sequences, 198 sequences (53%) were identified to phylum level as *Chloroflexi* Uncultured clones, with at least 99% percent identity. The rest of the ASVs (173; 47%) which were not assigned to genus level were assigned to family level include: *Rhodocyclaceae* (53), *Gemmataceae* (37), and 51 other families (Supplementary Figure S6).

Principal Coordinate Analysis (PCoA) of the beta diversity of water showed that the first two components explained 38% of the variance, primarily separating water from the farm in June and August (Supplementary Figure S7), although the *p*-value of the PERMANOVA was not significant (*p* = 0.383). The beta diversity of the water from the farm and the facility were grouped together over the remaining months.

#### 16S rRNA gene amplicon community profiling of environmental swabs

**Figure 6:**
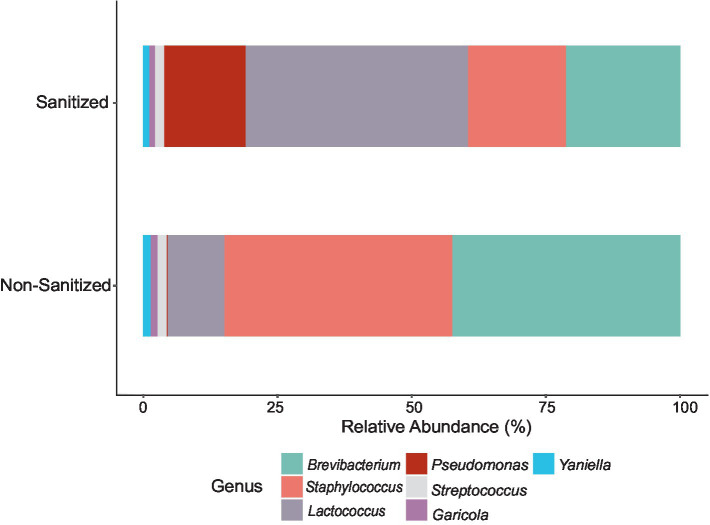
Bar plot of the relative abundance of genera obtained from 16S rRNA gene amplicon sequences of DNA extracted from sanitized versus non-sanitized swab samples of the cheese facility. Total ASVs were summed for each genus to determine the relative abundance within the respective sampling area.

Fifty-one samples contained adequate DNA for 16S rRNA gene sequencing out of 384 total samples from the entire sampling period (Supplementary Table S3). Of these 51 samples, 35 were obtained from the farm environment and 16 from the facility, ranging in number of reads from 1,016 to 140,744 (Supplementary Figure S3). Nine of the 16 swabs obtained from the facility were collected before sanitation, so they are presented separately (Figure 6; Supplementary Figure S8). *Brevibacterium* was the most abundant genus found in non-sanitized samples and was the second most abundant in sanitized samples. *Brevibacterium* and *Staphylococcus* were higher in abundance in samples taken before sanitation than after while the abundance of *Pseudomonas* and *Lactococcus* was higher in sanitized samples.

**Figure 7:**
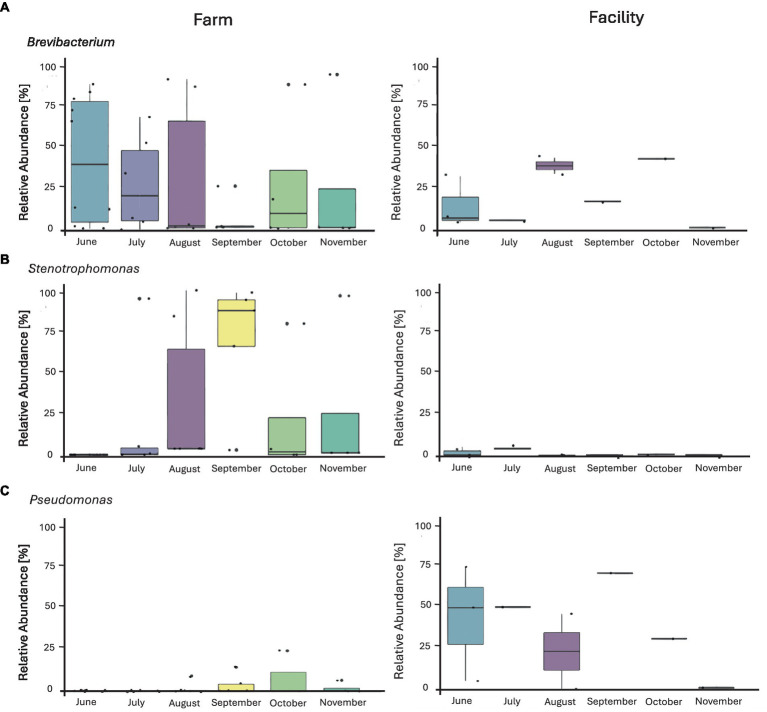
Box plots of the relative abundance of three major identified genera from environmental swabs obtained from the farm and cheese production facility (sanitized surfaces only) over 6 months of sampling. **(A)** *Brevibacterium*, **(B)** *Stenotrophomonas*, **(C)** *Pseudomonas*. The line on top of the whisker depicts the upper extreme (Max) then meets the upper quartile (Q3). The middle line is the median, followed by the lower quartile (Q1), meeting with the whisker to the lower extreme (min). The box itself is the interquartile range (IQR). Each individual sample is represented by a dot.

Prior to filtering, a total of 8,748 ASVs were obtained from swabs. Filtering was conducted at 20% prevalence and 0.01% relative abundance, resulting in 12 genera accounting for 236 ASVs. The 12 genera were identified as: *Brevibacterium, Delftia, Enhydrobacter, Garicola, Lactiplantibacillus, Lactococcus, Pediococcus, Pseudomonas, Staphylococcus, Stenotrophomonas, Streptococcus,* and *Yaniella*. The microbial composition of the swab samples from the farm environment was more diverse than that of the cheese production facility (Figure 7; Supplementary Figure S9). Genera which showed the greatest abundance were *Brevibacterium, Stenotrophomonas,* and *Pseudomonas*. In months where *Brevibacterium* was at the highest relative abundance on farm (June and August), there was greater relative abundance seen in swab samples obtained from the cheese facility. *Stenotrophomonas* was very abundant, particularly in September, in the swabs from the farm, but showed very low relative abundance in the cheese facility. *Pseudomonas* was more abundant in the cheese facility than the farm, where only very low relative abundance was seen in the later months of the sampling period (September–November). *Pseudomonas*, *Staphylococcus* and *Stenotrophomonas* were significant at 95% confidence interval (CI) by Tukey’s HSD when compared by location (farm versus facility).

**Figure 8:**
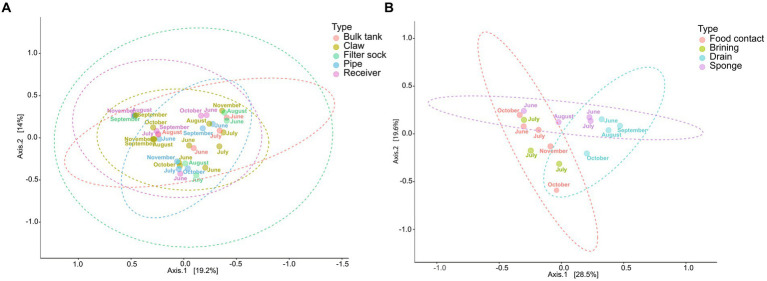
Principal coordinate analysis (PCoA) of the microbial beta diversity of environmental samples from the farm **(A)** and cheese production facility **(B)** using Bray-Curtis distance measures, and pairwise PERMANOVA for statistical significance (A: *p-value =* 0.735; B: *p-value =* 0.03).

The principal coordinate analysis (PCoA) of the beta diversity of the environmental swabs from the farm revealed no significant difference among sample types or months along axes 1 and 2 (*p* > 0.05 Figure 8A). In contrast, the facility showed three major sample groups (Figure 8B). Food contact and brine tank fell within the same ellipse. Drain samples were mostly distant from food contact surfaces, which was expected due to the dominance of *Pseudomonas* within the drain, but the lack of this genus on food contact surfaces. The beta diversity of sponge samples was spread between these two ellipses, as those obtained in June and July showed more similarity with the drain samples. No significant variation was found by farm sample type, but farm samples did differ significantly from the facility (Table 4). The variance explained by the first two coordinates for farm samples is approximately 33% in comparison to the facility which displayed approximately 48% of the variance accounted for by the first two components.

**Table 4:** Summary of pairwise PERMANOVA analysis of microbial beta diversity by sample location for swabs from the farm and facility obtained using the Bray-Curtis distance measurement.

Sample type	*R* ^2^	*p*-value
Claw/milk hose vs. Receiver	0.03	0.449
Milking system vs. Pipeline	0.04	0.416
Milking system vs. Filter sock	0.01	0.950
Milking system vs. Facility	0.17	0.001*
Receiver vs. Pipeline	0.08	0.442
Receiver vs. Filter sock	0.07	0.441
Receiver vs. Facility	0.27	0.002*
Pipeline vs. Filter sock	0.10	0.536
Pipeline vs. Facility	0.32	0.001*
Filter sock vs. Facility	0.25	0.010*

Multi-testing adjustment is based on the Benjamini-Hochberg procedure (FDR). Significance is denoted by an asterisk.

**Figure 9:**
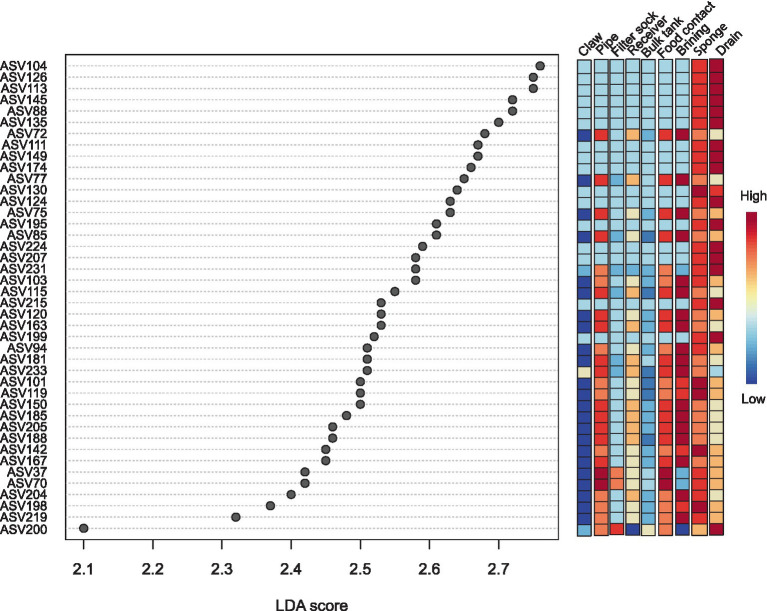
Linear discriminate analysis effect size (LEfSe) of all environmental swabs of mainly sanitized surfaces (except the brine tank, which was sanitized once in August) across farm and facility. The claw column combines data from the claw, cup, liner, short hose, proximal and distal hoses of the milking system. *P*-Value cut off = 0.1, and a log LDA score of 2.0 and over.

LEfSe analysis revealed that 42 out of 236 ASVs across both facilities showed significant differential abundance among sites with a Linear Discriminate Analysis (LDA) score of 2.0 or above (Figure 9). In the milking system composed of the milking cup liner, claw, short tube, proximal and distal milk hoses on farm (labelled claw in Figure 9) showed no significant ASVs. Only one ASV of mildly high significance was obtained from the filter sock and identified as *Garicola* (ASV 200; *Micrococcaceae*) and was the closest in abundance with the drain from the facility. The milk pipeline on the farm had 2 ASVs which were highly significant and identified as *Brevibacterium* (ASV 37, ASV 70). Food contact surfaces obtained from the facility did not have any significantly shared ASVs between all swab areas. Brine tank and rind wash cart samples contained 17 ASVs which were *Staphylococcus* (ASV 72, ASV 77, ASV 75, ASV 85, ASV 103, ASV 115, ASV 163, ASV 181, ASV 233, ASV 101, ASV 185, ASV 205, ASV 188, ASV 167, ASV 204, ASV 291) and *Pseudomonas* (ASV 199). Sponges showed 5 significant ASVs as *Staphylococcus* (ASV 119, ASV 150, ASV 167, ASV 194) and *Pseudomonas* (ASV 124). All *Staphylococcus* ASVs showed high identity with *S. equorum* (including uncultured clones). Finally, of the 42 ASVs identified, 16 were the most abundant in drains and composed of *Pseudomonas* (ASV 104, ASV 126, ASV 113, ASV 145, ASV 88, ASV 135, ASV 111, ASV 149, ASV 174, ASV 124, ASV 195, ASV 224, ASV 207, ASV 231, ASV 215, ASV 199) and one *Garicola* (ASV 200). *Pseudomonas* ASVs, which were highly prevalent in the drain, were also seen in sponge samples and once within the brine tank and rind washing cart samples.

### Consistency of genera over farm and cheese processing facility

**Figure 10:**
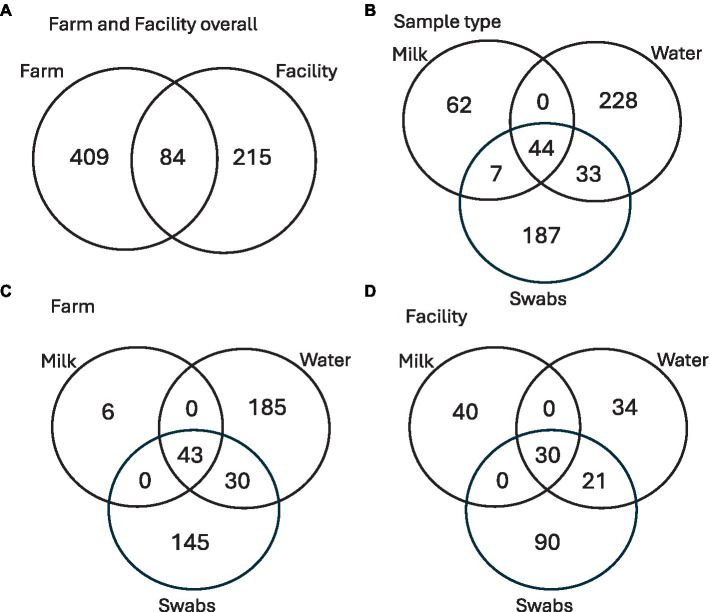
Venn diagram of **(A)** shared and unique ASVs found on farm and facility; **(B)** shared and unique ASVs found within each sample type (milk, water, and swabs); **(C)** shared and unique ASVs of the sample types obtained from the farm; **(D)** shared and unique ASVs of the sample types obtained from the facility.

Two actinomycete genera identified as *Brevibacterium* and *Yaniella* were shared between all sample types of milk, water, and swabs (Figure 10; Table 5). Milk showed two unique genera, both *Actinomycetota* (*Knoellia* and *Rothia*) as well as two shared ASVs identified as *Kocuria* (*Actinomycetota*) and *Paracoccus (Alphaproteobacteria)*. Both on the farm and in the facility, milk contained unique ASVs that were not seen in any other sample types (Figure 10). Water showed the greatest diversity of genera, sharing six genera with swab samples, identified as *Garicola, Stenotrophomonas, Lactococcus, Pseudomonas, Streptococcus* and *Staphylococcus*. Water also contained six genera which were unique to the environment: *Brachybacterium, Corynebacterium, Gallionella, Lactobacillus, Novosphingobium* and *Sulfuricella*.

**Table 5:** Summary table of common (C), unique (U), and total (T) ASVs found across milk, water and swabs for both farm and cheese processing facility at 0.01% relative abundance (RA) and 20% prevalence in samples.

	Milk					Water					Swab				
Genus	# of ASVs			RA [%]		# of ASVs			RA [%]		# of ASVs			RA [%]	
	C	U	T	Farm	Facility	C	U	T	Farm	Facility	C	U	T	Farm	Facility
*Brachybacterium*	0	0	0	0	0	0	32	32	3	5	0	0	0	0	0
*Brevibacterium*	15	1	16	16	19	40	74	114	41	21	40	1	41	14	28
*Corynebacterium*	0	0	0	0	0	0	24	24	4	1	0	0	0	0	0
*Delftia*	0	0	0	0	0	0	0	0	0	0	0	6	6	3	0
*Enhydrobacter*	5	2	7	2	10	0	0	0	0	0	5	0	5	2	0
*Garicola*	0	0	0	0	0	33	6	39	22	7	3	0	3	1	2
*Knoellia*	0	2	2	2	3	0	0	0	0	0	0	0	0	0	0
*Kocuria*	27	5	32	49	36	0	0	0	0	0	0	0	0	0	0
*Lactobacillus*	0	0	0	0	0	17	2	19	15	1	0	0	0	0	0
*Lactococcus*	0	0	0	0	0	24	0	24	2	58	37	1	38	12	26
*Pediococcus*	0	0	0	0	0	0	0	0	0	0	0	19	19	10	0
*Paracoccus*	21	5	26	26	31	0	0	0	0	0	0	0	0	0	0
*Pseudomonas*	0	0	0	0	0	0	0	0	0	0	3	17	19	1	19
Staphylococcus*	4	67*	71	2	1	28	0	28	3	2	22	0	22	1	23
*Stenotrophomonas*	0	0	0	0	0	0	0	0	0	0	33	44	77	56	1
*Yaniella*	3	0	3	5	2	4	16	20	11	5	3	0	3	1	1

*Staphylococcus was absent at 20% prevalence and 0.1% RA in milk samples obtained from the farm and facility but was found at 0.01% RA in milk. “0” denotes no identified genera for that location or sample type.

For comparison, the common, unique, and total ASVs are shown for all sample types (Table 6). Water and swabs shared two genera, *Lactococcus* and *Staphylococcus*, which were not shared with milk (Table 6). Milk and swabs shared one common genus, which was identified as *Enhydrobacter (Alphaproteobacteria).* Finally, milk and water did not share any genera at 20% prevalence. A total of 171 ASVs belonging to *Brevibacterium* were found across all sampling types, with 40 shared ASVs, while out of 26 ASVs of *Yaniella*, 4 were shared across all sampling types. *Staphylococcus* was seen in all sampling types (milk, water, and swabs) and was prevalent in >20% of samples of water and swabs but not prevalent in milk. When present, *Staphylococcus* relative abundance was below 0.1%, but above 0.01%. As an example of transfer, one significant *Staphylococcus* ASV 233 was found from the claw all the way to the brine tank and sponges of the facility. BLAST of shared *Staphylococcus* sequences showed 100% query coverage and 99.57% percent identity with partial sequences of *Staphylococcus equorum*.

**Table 6:** Top taxa shared between sample type.

Genera	Total number of ASVs	Shared ASVs
*Lactococcus*	62	20
*Staphylococcus*	49	13
*Enhydrobacter*	14	7
*Brevibacterium*	171	40
*Yaniella*	26	4

Total #ASVs indicate how many ASVs were within genera, whereas shared ASVs indicate the number of common ASVs of those genera shared between milk, water and swabs.

The alpha diversity of filtered samples showed no significant differences between month by Mann–Whitney (*p* > 0.05) (Supplementary Figure S10). In comparison, unfiltered swabs samples showed one significant comparison of November versus September (*p* = 0.05).

## Discussion

The objective of this study was to determine the persistence and composition of post-cleaning residual contamination of a cheese production facility using a sole farm milk source over a six-month period in relation with the microbial communities of raw milk and water. Investigating the shared bacteria between systems can aid in determining whether there is consistent transfer of bacteria between farm and processing facility. In previous studies, milk has been a driving factor in the temporal and spatial variation observed within Cheddar cheese plants (Johnson et al., 2021), but this could be attributed to obtaining milk from multiple sources. The farm sampled for this study is the sole source of bovine milk for an adjacent processing facility, so the variation can be attributed to conditions along a single farm to facility site. For the duration of the study, a core microbiome at the phylum level (70–80% prevalence) consisting of *Actinomycetota* (*Brevibacteriaceae* and *Micrococcaceae*) showed the wide-spread stability of this phylum in milk, water and swabs across the farm and cheese-making facility. Genera which were observed at lower prevalence (<20%) highlight the spatial variability across the locations and sampling environments. *Actinomycetota* (synonym *Actinobacteria*) have been one of the most frequently identified phyla in relation to washed-rind cheese production and a dominant phylum found in milking systems (Quijada et al., 2018; Weber et al., 2019). In soil–plant ecosystems, *Actinobacteria* play an important role in degrading organic matter and mobilizing nutrients and minerals (Boubekri et al., 2022). As their proposed biotechnological uses as biocontrol and biofertilizers become more popular in agriculture, the probability of transfer of *Actinobacteria* to the dairy production and processing systems may increase. This warrants further investigation into whether the diversity of *Actinobacteria* will change on farm and how this may affect product quality.

Milk; raw or pasteurized, is a large contributing factor of microbes to cheese production (Johnson et al., 2021; Sun and D’Amico, 2021), accumulating microbes as it moves through the teat canal and through the milking system (Frétin et al., 2018; Du et al., 2020). Improper or poorly managed bedding materials are one of the most significant areas where environmental mastitis pathogens can be introduced to lactating cows (Ray et al., 2022). In the current study, the culture-dependent methods focused on selection for lactose-utilizing bacterial species, yeasts and molds, which may exclude other bacterial types, with no enrichment for pathogenic or spoilage bacteria. Therefore, we cannot conclude on contamination of milk with specific pathogens or spoilage agents such as spore-forming bacteria. The culture-independent method of 16S rRNA gene amplicon sequencing can detect dominant taxa (not discriminating dead from alive) but may also miss low frequency contaminants. However, combining both methods can provide complementary information on the major types of microorganisms that resist the cleaning process and are shared between the farm and cheese-making facility. The consistent bacteria of milk found in this study revealed a high prevalence of *Brevibacterium* spp. which has been most associated with the rind of smear ripened and washed-rind cheeses and is commonly found in soils, sediment, and seawater (Forquin-Gomez et al., 2014; Pham et al., 2017; Erkmen, 2022). The origin of *Brevibacterium* in raw milk is relatively unknown, but with the wide variety of isolates collected across many substrates, especially in soil, the transfer of bacteria into the dairy environment may be through bedding, feed, or water. While *Brevibacterium linens* is the most widely used ripening adjunct culture for these types of cheeses (Irlinger and Mounier, 2009), the wide-spread occurrence and diversity of this genus in the current farm and cheese facility may contribute positively to the natural development of rind color.

The microbial load of milk did not significantly change after transportation from the farm bulk tank to the milk holding tank at the cheese facility, reflecting the short holding time and proper cooling. For this study, milk was not kept in the farm bulk tank for longer than 24 h before being transferred in a truck from the farm bulk tank to the cheese production facility, where it could be held for up to 24 h. The proximity of the facilities, the use of correct prescribed cooling and holding temperatures, proper cooling and cold storage of milk facilitates the maintenance of good microbial quality (O’Connell et al., 2016; Paludetti et al., 2018). A comparison of genera in milk revealed a higher abundance of *Enhydrobacter* after transportation to the facility holding tank. The increase of *Enhydrobacter* and its origin within the dairy setting are still unknown, but studies conducted mainly in forensics have found *Enhydrobacter*, as well as *Staphylococcus*, to be major taxa found on the palms of humans (Park et al., 2017). No *Enhydrobacter* was found in swab samples obtained from the bulk tank and milk holding tank at the facility, suggesting the incorporation of the bacteria into the milk through contact with equipment manipulated by personnel during transport. Further studies of the tanker truck, particularly the hose, are necessary to evaluate the risk of contamination of raw milk from this source.

Despite the proximity of the farm to the cheese production plant, independent wells and water piping systems may contribute to the variation in microbial profiles between water sampling sites. During periods of heavy rainfall, well water safety can be compromised, leading to higher microbial accumulation in water (Powers et al., 2023). During June and August of 2022, significant rainfalls ranged from 10 to 15 mm per day (Government of Canada, 2023). The increase in rainwater, potentially adding to the groundwater composition, may explain the variability within those months compared to others. Chlorine is used to treat tap water at both facilities. Although there are no studies showing the exact chlorine resistance of *Brevibacterium*, one study suggests that association with chlorine-resistant bacteria such as *Kocuria* sp. and *Staphylococcus sciuri* can generate a protective effect for each other but culturable strains of *Brevibacterium* were undetectable (Leriche et al., 2003). *Staphylococcus* was found in water and swabs, and can be commonly found in well water that is improperly treated (Silva et al., 2020). Proper water supply treatment with chlorine is sufficient for controlling *Staphylococcus aureus* in water (Santos et al., 2020). The 16S rRNA analysis does not distinguish the viable state of bacteria unless cell pellets are treated with a dye such as propidium monoazide before DNA extraction. Propidium monoazide (PMA) treatment is an additive that distinguishes living and dead or permeable bacteria (Golpayegani et al., 2019). Further studies are needed to conclude on the state of the bacteria found within water samples that may not be culturable on plates to fully understand the impact they may have on the microbial community on farms and during processing.

*Brevibacterium* spp. within this study showed consistency throughout all sample types, so it is either able to adhere to equipment or join biofilms formed by other bacteria, resisting the cleaning process and transferring from farm to facility. Most studies on the ability of *Brevibacterium* to form biofilms have been in relation to the rhizosphere, where *Brevibacterium* is a native biofilm former although a tested strain of *Brevibacterium frigoritolerans* did not form biofilm on high-density polyethylene (Liu et al., 2013; Ansari and Ahmad, 2019). As shown by Leriche et al. (2003), *Brevibacterium linens* in monoculture showed very weak ability to synthesize extracellular polysaccharides and proteins when subject to chlorinated alkaline solutions, similar to those used in cheese production facilitates. Hoses in the milking system, such as the short tube, proximal milk hose and distal milk hoses, are commonly made from a plastic or silicone material, which may be an area of interest for the ability of *Brevibacterium* and other bacteria to form biofilms, but there have been no studies regarding the formation of biofilms on such materials. Stainless steel is another common material used in milking systems and cheese production facilities. Further investigation into the ability of *Brevibacterium* to form biofilms within these types of environments (PVC and stainless steel) is needed to elucidate their persistence in the milking system and transfer into raw milk.

*Stenotrophomonas* spp. has been frequently isolated from raw milk and raw milk products (El-Prince et al., 2019). This genus has many adaptive properties allowing for growth at low temperatures and during food preservation (El-Prince et al., 2019), but does not possess any mechanisms for heat tolerance and is therefore generally eliminated during pasteurization (Ranieri et al., 2009). The elimination after pasteurization explains the very low relative abundance found within the cheese production facility when using pasteurized milk for production, while *Stenotrophomonas* was more abundant in the milking system on farm. It appears that the routine for cleaning the milking system seems to result in residual contamination by *Stenotrophomonas* spp.. Due to the limitations of amplicon sequence variant databases, it is not possible to achieve reliable species level identification using the 16S rRNA gene amplicon. Further studies of *Stenotrophomonas* are required to determine whether biofilm formation leads to reducing the effectiveness of cleaning and sanitation agents within the farm environment (Isom et al., 2022). In comparison, *Pseudomonas* is of concern in all water studies due to the ability to form biofilms (Mann and Wozniak, 2012) in sections of drains and become resistant to low levels of chlorine (Navarro-Noya et al., 2013; Mao et al., 2018). In the current study, *Pseudomonas* was indeed more prevalent in the drain of the facility than on farm. The growth of *Pseudomonas* in drains can be expected and explains the dominance of this genus in the swabs obtained from the drain within the facility. The temporal variation of *Pseudomonas* within the drain can be explained by bi-weekly cleaning when the drain catch is removed and thoroughly cleaned to prevent the accumulation of bacteria within this drain and, thus, within the starter culture room.

Over 3 days of production, Johnson et al. (2021) found temporal variation with very few genera shared between milk samples and environmental samples in three Cheddar cheese facilities using multiple sources of milk, where sampling was conducted before sanitation. The current longitudinal study over 6 months in one facility showed no significant changes in viable plate counts of milk over the sampling period, but few genera were shared between farm and facility. Of the shared genera between milk, water and environment found in this study, *Brevibacterium* and *Yaniella* were the only genera which showed continuity between all ecosystems. In the Johnson et al. (2021) study, *Lactococcus* was the only genus which was present in all sample types, including equipment before sanitation. *Lactococcus* found in water and swab samples in this study are congruent with Johnson et al. (2021) and can be attributed to the use of starter culture in cheese production but this genus is also commonly found in raw milk (Bokulich and Mills 2013; Quigley et al., 2013; Johnson et al., 2021). In the current study, *Lactococcus* genera were obtained in high abundance on planks in aging room 1, which houses freshly made cheeses for 5 months. After 5 months, cheeses are moved into aging room 4, where genera such as *Lactococcus* is replaced in abundance by yeasts and molds, congruent with aging (Wolfe et al., 2014). Instead of *Lactococcus* found consistently before sanitation, the effect of cleaning revealed genera such as *Brevibacterium* and *Yaniella*, which were consistent across all sample types afterwards. Each sample type revealed unique genera within their respective environment.

*Staphylococcus* was only found sporadically in milk samples. *Staphylococcus* ASVs were identified as *S. equorum,* which is common on washed-rind cheeses. This food-grade S*taphylococcus* species has been found in aged brines at up to 5 log CFU per mL (Mounier and Coton, 2022). This bacterium may have been transferred from the farm environment to the facility but could also be tracked in by personnel. As they are halotolerant, *S. equorum* may potentially contribute to cheese ripening and the control of mold growth (Kastman et al., 2016; Mounier and Coton, 2022). In the current study, no bacterial genera were present in over 20% of samples across the two sites, evidence for a high level of variation of the milk profile from month to month, suggesting the sporadic nature of contamination. The analysis of sanitized versus non-sanitized equipment within the facility showed a few prevalent genera, but large differences in the relative abundance of each genus. *Brevibacterium* and *Staphylococcus* decreased by at least 50% in abundance after sanitation, but they were not completely eliminated, while *Lactococcus* and *Pseudomonas* greatly increased in relative abundance post-sanitation. This supports their relatively greater ability to withstand cleaning compounds, perhaps due to biofilm formation, and contribute to the in-house microbiota. Reviews conducted by Marchand et al. (2012), Vlková et al. (2008), and Flint et al. (2020), highlight the increased resistance to sanitation acquired by multispecies biofilms and the risk of subsequent spoilage from spoilage bacteria potentially protected within the consortium. Increasing the frequency of in-depth cleaning of the drain from bi-weekly to weekly could alleviate some of the residual contamination that was found. Within the study by Johnson et al. (2021), the day-to-day microbiota on surfaces that were presumably not sanitized included bacterial species such as *Acinetobacter*, *Escherichia*, *Klebsiella*, and *Enterococcus*, which are common dairy facility biofilm formers (Cherif-Antar et al., 2016) within a facility which had been in operation for 10–15 years. Johnson et al. (2021) hypothesized that this increased bacterial diversity on the food contact surfaces was due to the longer operation time than the two other facilities. In the current study, the facility had been in operation for approximately 10 years but lacked the presence of these common dairy facility biofilm formers, which may reflect the effectiveness of the sanitation procedure, particularly of food contact surfaces. However, the consistent presence of a few shared genera between farm and facility suggests either the regular constant entry or the ability to adhere irreversibly to abiotic surfaces within the equipment at each location, as they were obtained after sanitation.

Further studies should include whole genome sequencing of *Brevibacterium* spp. and *S. equorum* found on the farm and within the cheese facility to investigate congruent strains between the two facilities. This can provide further insight into the species and even strain-level traceability of these taxa, which can potentially contribute positively to cheese ripening. The biofilm formation potential of *Brevibacterium* spp. on substrates such as stainless steel and PVC would provide insight into the ability to remain within the dairy production and processing systems. A further investigation into the yeast and mold community would enhance the understanding of the role fungi play in the development of the cheese rind and the succession of the microbiota as the cheese ages (Wolfe et al., 2014).

## Conclusion

In conclusion, this six-month study revealed the overall stochastic nature of the microbiota composition between one farm to a sole milk source production facility over time and space, given the low prevalence and abundance of the majority of taxa identified. Most studies which tracked dairy microbial ecosystems in the past have focused on multiple farms or short-term studies, whereas this study looked at a singular farm longitudinally over a six-month period. Because of the sole milk source, this study demonstrates which microbes are more likely to transfer from farm to facility (*Actinomycetota*), and which may be enriched during transport and in the facility (*Pseudomonas* spp.). Milk showed the most stability in viable plate count over the duration of the study, even after travel between facilities, supporting the advantage of sole milk sources and the importance of proper storage and temperature. The level of post-cleaning residual contamination was generally higher in the milking system on farm than in the facility, and the consistently high microbial load of the milking hoses is of particular concern for subsequent downstream contamination. *Pseudomonas*, *Lactococcus*, *Staphylococcus*, and *Brevibacterium* remain on some surfaces in the facility between cleaning cycles. However, shared sequences of a limited number of genera did occur from farm to facility. Amplicon sequence variant analysis (ASV) revealed *Brevibacterium* and *Yaniella* (*Actinomycetota*) as shared genera between the farm and cheese-making facility as well as between the milk, water, and swabs. These two bacterial genera belong to groups that have been associated with cheese ripening. Unique ASVs highlight the intra-genus diversity at each sample location that should be further investigated.

## Data Availability

The datasets for this study are available in the Borealis Agri-Environmental Research Data Repository of the University of Guelph at https://doi.org/10.5683/SP3/G2DU0Q.
