# Supplementary material for: Tracking the microbial communities from the farm to the processing facility of a washed-rind cheese operation
Source: Front Microbiol. 2024 Aug 29;15:1404795. doi: 10.3389/fmicb.2024.1404795 (PMC11390512; doi:10.3389/fmicb.2024.1404795)
Supplement: Supplementary file 1 [file Data_Sheet_1.PDF]

Table S1. Milk sample DNA which had adequate amounts (~5 ng/µL) for 16S rRNA gene amplicon sequencing.

Sample	Location	Month of Collection	DNA present
Milk	Farm	June	
	Farm	June	X
	Farm	July	X
	Farm	July	
	Farm	August	X
	Farm	August	X
	Farm	September	
	Farm	September	
	Farm	October	
	Farm	October	X
	Farm	November	
	Farm	November	
	Facility	June	
	Facility	June	X
	Facility	July	X
	Facility	July	X
	Facility	August	X
	Facility	August	X
	Facility	September	
	Facility	September	X
	Facility	October	X
	Facility	October	X
	Facility	November	
	Facility	November	X

Table S2. Well water samples with adequate DNA amounts (~5 ng/µL) for 16S rRNA gene amplicon sequencing.

Sample	Location	Month of Collection	DNA present
Water	Farm	June	X
	Farm	June	
	Farm	July	X
	Farm	July	X
	Farm	August	X
	Farm	August	X
	Farm	September	
	Farm	September	
	Farm	October	X
	Farm	October	X
	Farm	November	
	Farm	November	
	Facility	June	X
	Facility	June	
	Facility	July	
	Facility	July	
	Facility	August	
	Facility	August	X
	Facility	September	
	Facility	September	
	Facility	October	
	Facility	October	X
	Facility	November	
	Facility	November	

Table S3. Environmental samples with adequate DNA amounts (~5 ng/µL) from farm and cheese processing facilities for 16S rRNA gene amplicon sequencing.

Location	Farm or Facility	DNA present (at least one month)
Milking cup liner (1)	Farm	X
Short tube (1)	Farm	X
Milking claw (1)	Farm	X
Proximal milk hose (1)	Farm	X
Distal milk hose (1)	Farm	X
Filter sock	Farm	X
Milking pipeline 1-4	Farm	X
Receiver group	Farm	X
Swing line	Farm	X
Bulk tank	Farm	X
Tanker hose	Facility	
Milk tank	Facility	
Line to pasteurizer	Facility	
Exit valve	Facility	
4000 L vat	Facility	
400 L vat 1*	Facility	X (n=1)
400 L vat 2*	Facility	X (n=1)
1500 L vat	Facility	
Whey exit	Facility	
Stirring blade	Facility	
Curd cutter	Facility	
Draining table	Facility	
Cheese mould	Facility	
Brine tank*	Facility	X (n=2)
Plank in aging room 1	Facility	
Plank in aging room 4	Facility	
Rind washing cart	Facility	X (n=1)
Cheese cutting blade*	Facility	X (n=1)
Cutting table*	Facility	X (n=2)
Lifting platform	Facility	X (n=1)
Shelves	Facility	
Racks	Facility	
Wall	Facility	
Floor	Facility	X (n=3)
Drain*	Facility	X (n=4)

Notes:
* Samples denoted with an asterisk (*) were surfaces within the cheese processing facility which were sampled before complete sanitation for at least one sample and were analyzed separate.
(1) Samples were pooled for 16S rRNA gene amplicon sequencing, designated as “Claw”.

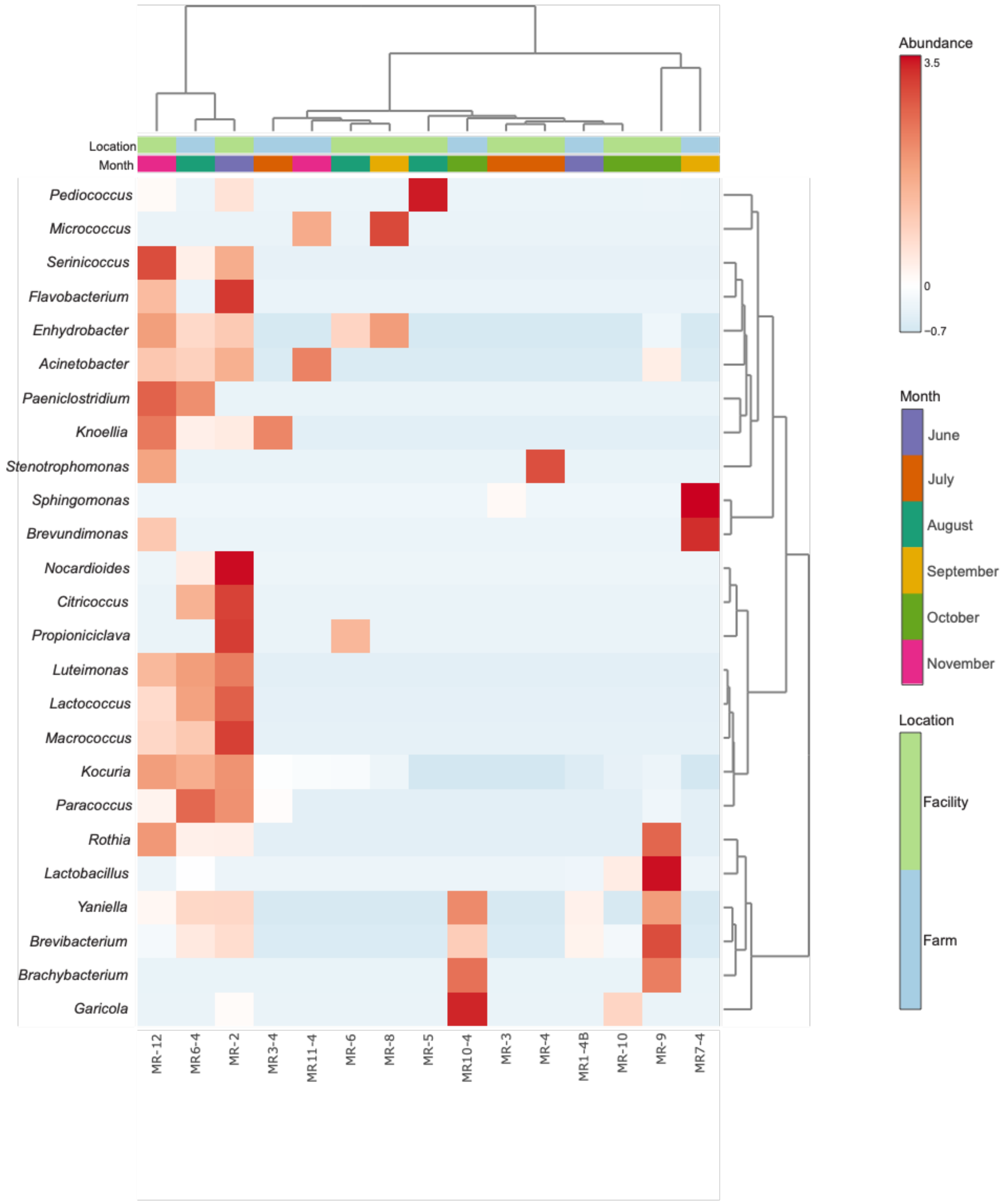

Figure S1. Heat map of the relative abundance of genera found by 16S rRNA gene amplicon sequencing of the DNA extracted from raw milk obtained from the farm and cheese processing facilities. Samples are standardized and normalized, clustering performed by Euclidean distance and Ward method.

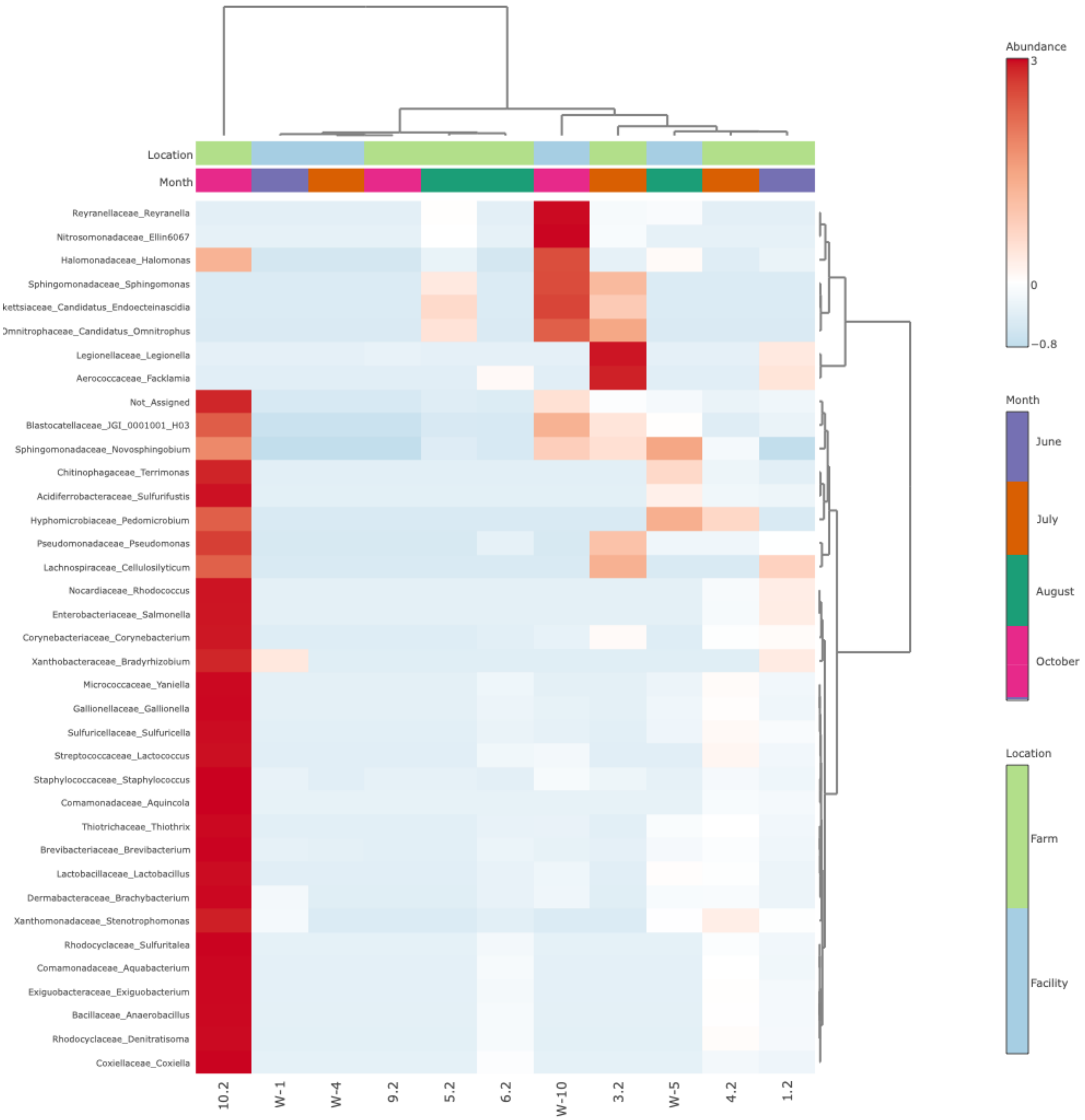

Figure S2. Heat map of the relative abundance of genera found by 16S rRNA gene amplicon sequencing of DNA extracted from tap water samples obtained from the farm and cheese processing facilities. Samples are standardized and normalized, clustering performed by Euclidean distance and Ward method.

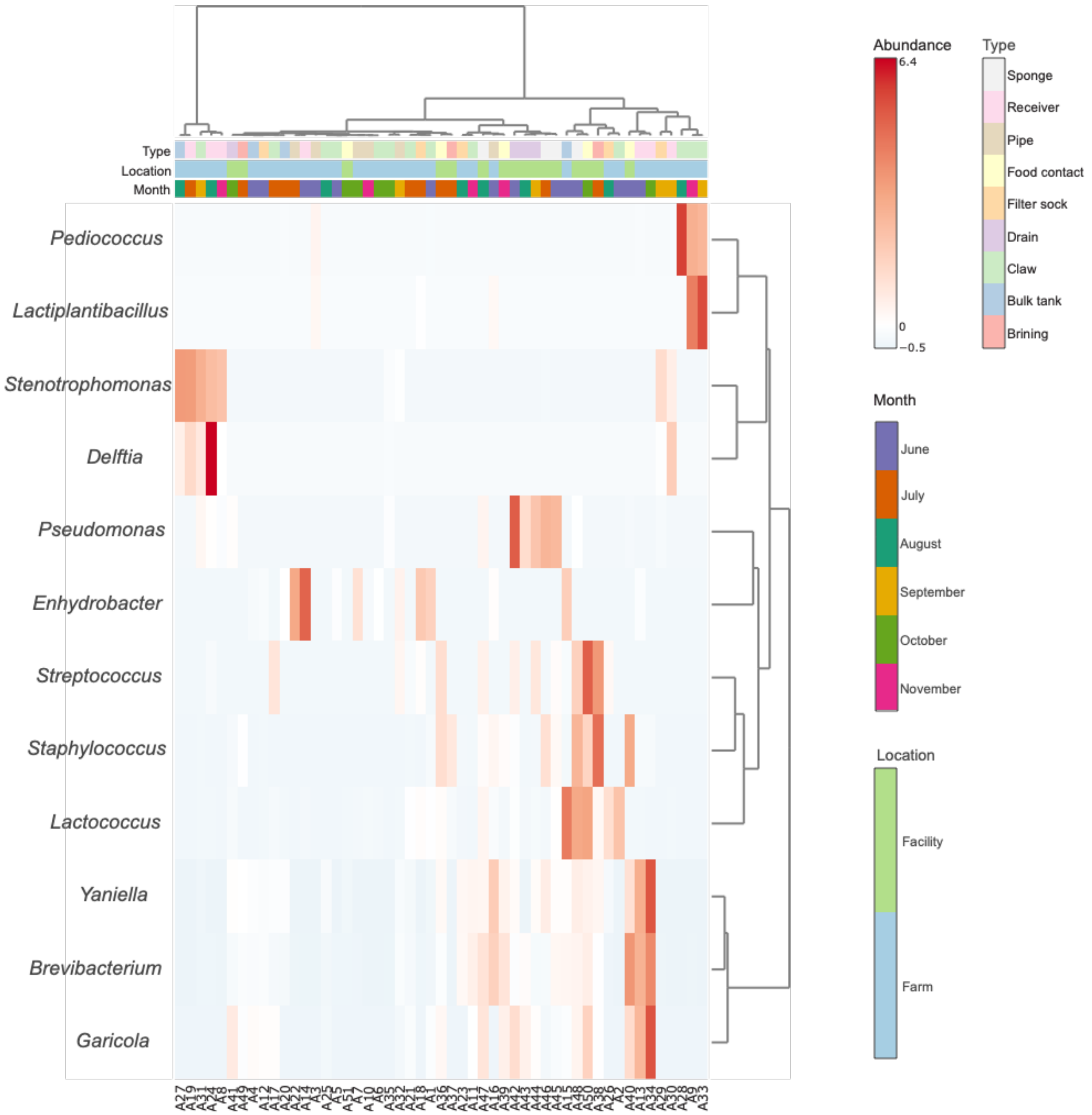

Figure S3. Heat map of the relative abundance of genera found by 16S rRNA gene amplicon sequencing of DNA extracted from environmental swabs obtained from the farm and cheese processing facilities. Samples are standardized and normalized, clustering performed by Euclidean distance and Ward method.

Table S4. Metadata of samples within Figure S3. A- codes represent configuration within Figure S3, sample codes denote codes used within the study represented by location, type and month

A- Code	Sample code	Location	Type	Month
A1	1.10	Farm	Claw	June
A2	1.12	Farm	Claw	June
A3	1.26	Farm	Pipe	June
A4	1.6	Farm	Bulk tank	June
A5	1.8	Farm	Claw	June
A6	10.MS	Farm	Claw	October
A7	10.P	Farm	Pipe	October
A8	12.30	Farm	Receiver	November
A9	11.MS	Farm	Claw	November
A10	11.P	Farm	Pipe	November
A11	12.MS	Farm	Claw	November
A12	2.18	Farm	Filter sock	June
A13	2.28	Farm	Receiver	June
A14	2.30	Farm	Receiver	June
A15	2.6	Farm	Bulk tank	June
A16	2.P	Farm	Pipe	June
A17	3.MS	Farm	Claw	July
A18	4.18	Farm	Filter sock	July
A19	4.30	Farm	Receiver	July
A20	4.6	Farm	Bulk tank	July
A21	4.MS	Farm	Claw	July
A22	4.P	Farm	Pipe	July
A23	5.18	Farm	Filter sock	August
A24	5.30	Farm	Receiver	August
A25	5.MS	Farm	Claw	August
A26	6.18	Farm	Filter sock	August
A27	6.6	Farm	Bulk tank	August
A28	6.MS	Farm	Claw	August
A29	7.18	Farm	Filter sock	September
A30	6.30	Farm	Receiver	August
A31	7.MS	Farm	Claw	September
A32	7.P	Farm	Pipe	September
A33	8.MS	Farm	Claw	September
A34	9.30	Farm	Receiver	October
A35	9.MS	Farm	Claw	October
A36	B.4	Facility	Food contact	July
A37	BT.3	Facility	Brining	July
A38	BT.4	Facility	Brining	July
A39	CT.11	Facility	Food contact	November
A40	CT.2	Facility	Food contact	June
A41	DDL.10	Facility	Drain	October
A42	DDL.2	Facility	Drain	June
A43	DDL.6	Facility	Drain	August

A44	DDL.7	Facility	Drain	September
A45	DF.2	Facility	Sponge	June
A46	DF.4	Facility	Sponge	July
A47	DF.6	Facility	Sponge	August
A48	DLP.2	Facility	Sponge	June
A49	RW.3	Facility	Brining	July
A50	V41.10	Facility	Food contact	October
A51	V42.10	Facility	Food contact	October

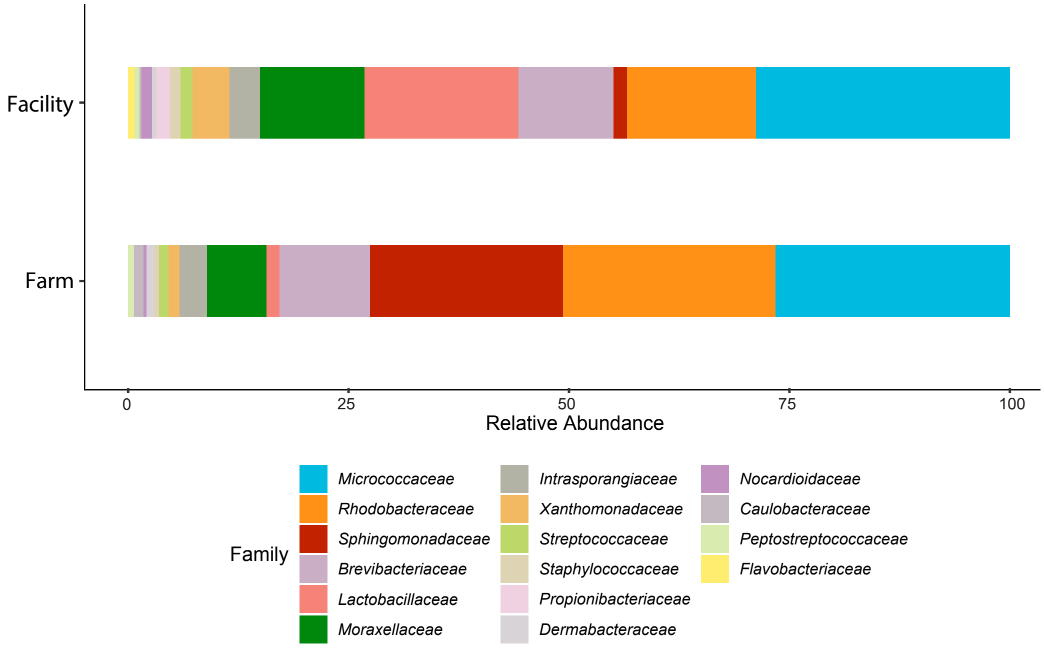

Figure S4. Stacked bar plot of family level analysis of the relative abundance of 16S rRNA gene amplicon sequences from DNA extracted from milk samples obtained from the farm and facility.

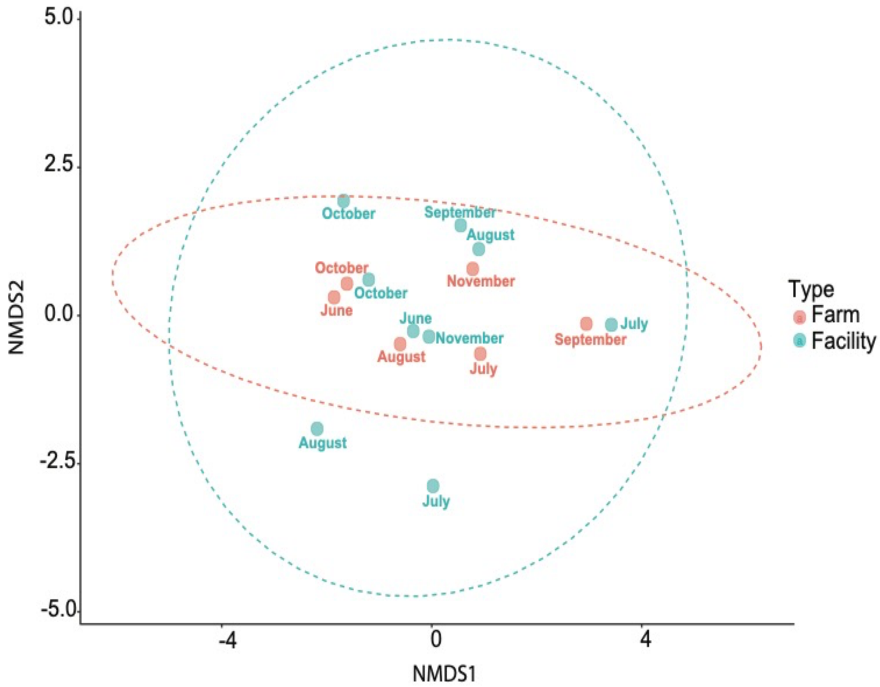

Figure S5. Non-metric multidimensional scaling (NMDS) of the beta diversity of the microbial community of milk samples obtained from farm and facility, using the Bray-Curtis distance method and pairwise PERMANOVA (*p-value* = 0.776) in R studio and Microbiome Analyst.

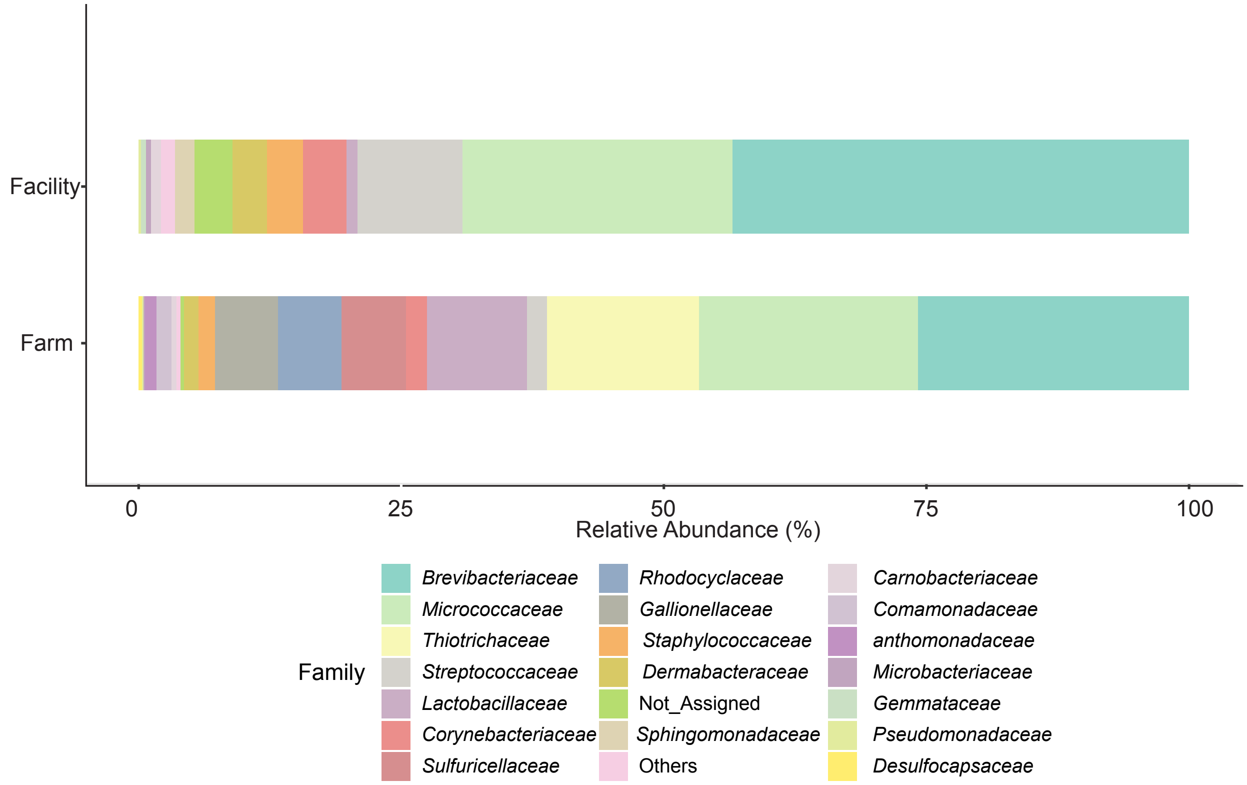

Figure S6. Stacked bar plot of family level analysis of the relative abundance of 16S rRNA gene amplicon sequences from DNA extracted from water samples obtained from the farm and facility.

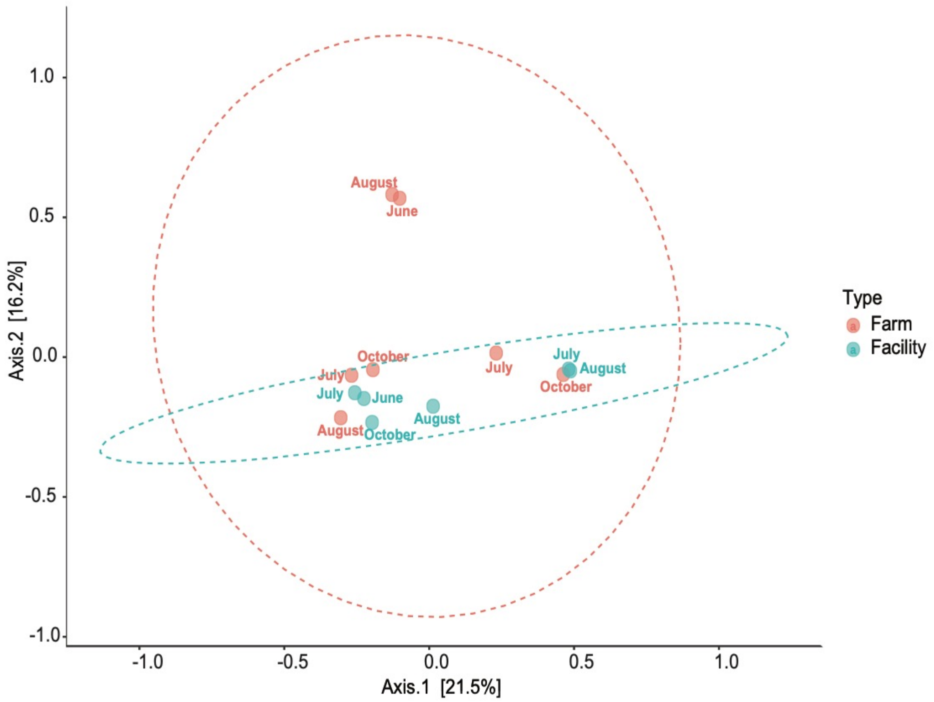

Figure S7. Principal Coordinate Analysis (PCoA) of microbial diversity of water from the farm and cheese production facility using Bray-Curtis distance measures, and pairwise PERMANOVA (*p-value* = 0.383).

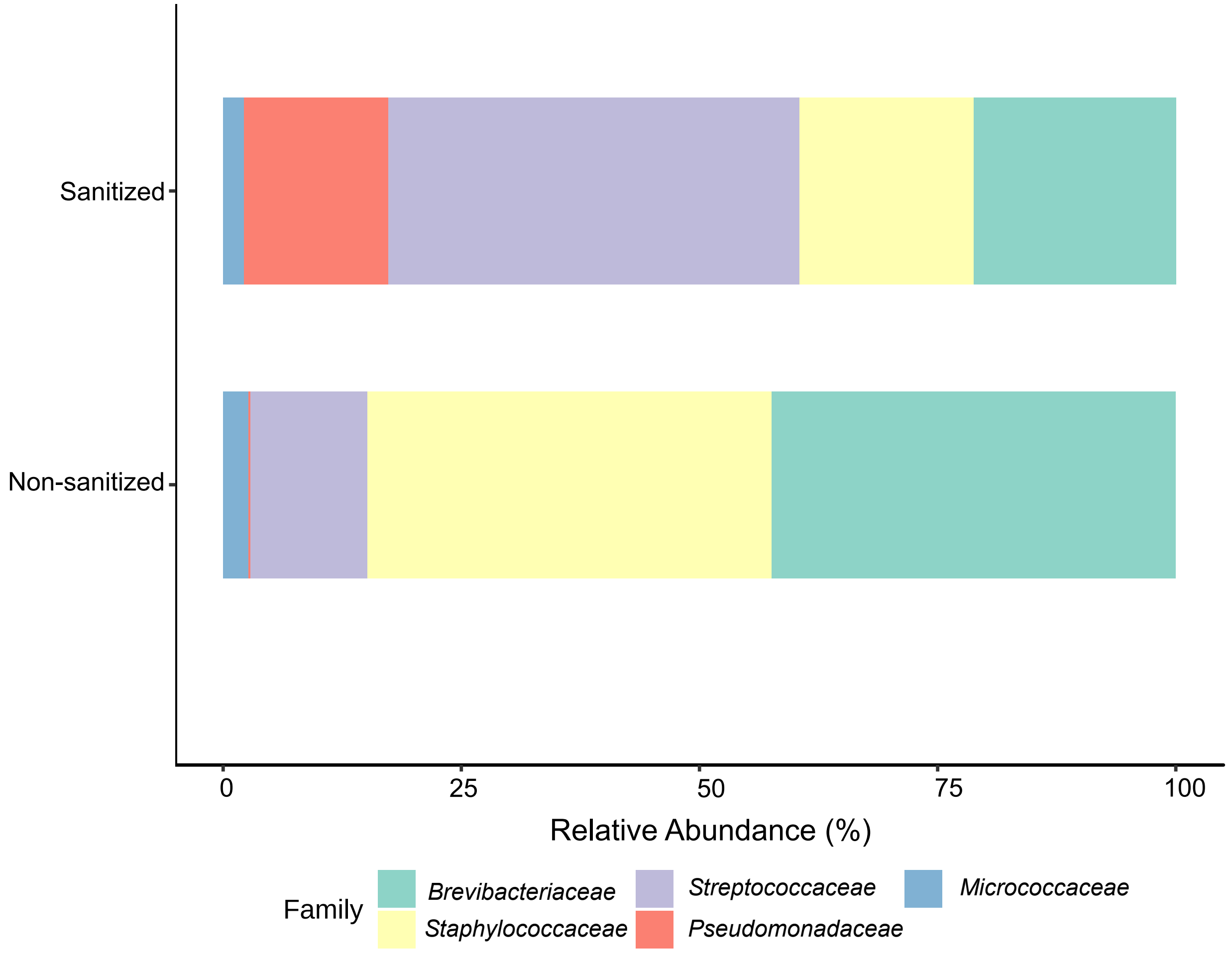

Figure S8. Stacked bar plot of family level analysis of the relative abundance of 16S rRNA gene amplicon sequences from DNA extracted from environmental swab samples obtained from the farm and cheese facility separated by sanitized (n=7) and non-sanitized samples (n=9).

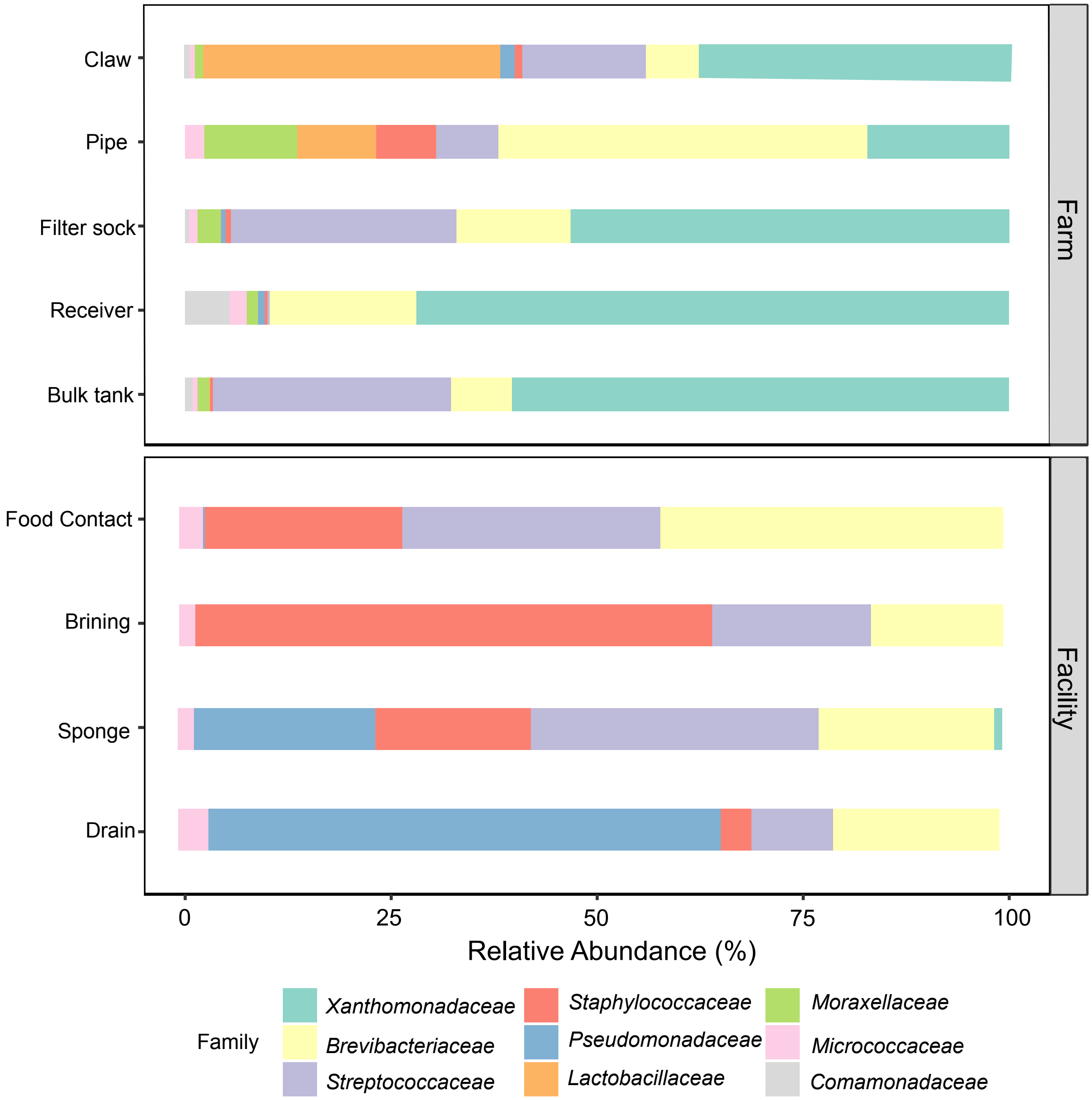

Figure S9. Stacked bar plot of family level relative abundance of 16S rRNA gene amplicon sequences from DNA extracted from environmental swab samples obtained from the farm and facility. The claw combines the claw, cup, liner, short hose, proximal hose and distal hose.

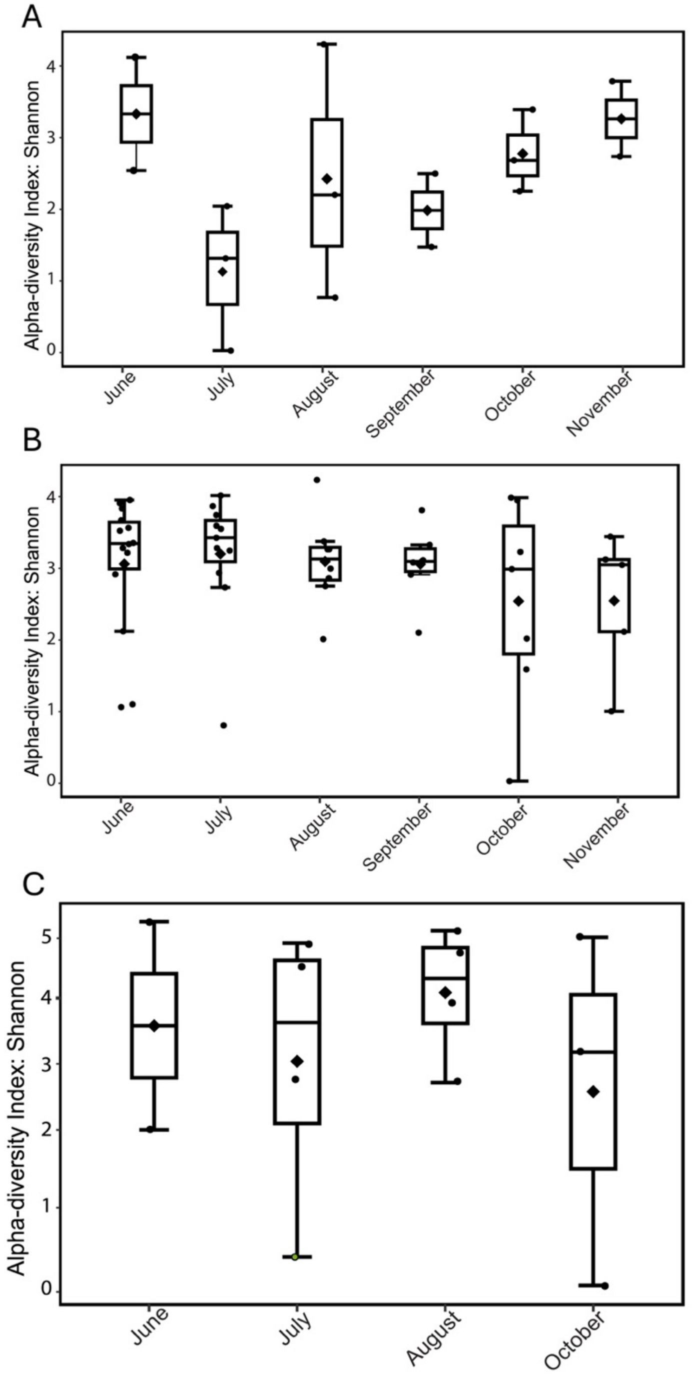

Figure S10. Shannon alpha diversity of milk, water and swab samples filtered at 0.01% relative abundance. (**A**) denotes milk samples, (**B**) swabs, and (**C**) water samples. No samples showed significant difference (P> 0.05) by post-hoc pairwise comparisons; Mann-Whitney test for each pair, adjustments were based on the Benjamini-Hochberg procedure (FDR). A comparison of filtered (0.01% RA) and non-filtered sample sets showed one significant value in swabs when comparing November vs. September (*p-value*= 0.05 by the Mann-Whitney pairwise comparison test). The line on top of the whisker depicts the upper extreme (Max), which meets the box of the

upper quartile (Q3). The middle line is the median, followed by the box with the lower quartile (Q1), then the whisker to the lower extreme (min). The box itself is the interquartile range (IQR). The diamond represents the mean.
